# IDH1-Associated m6A Methylation Is Linked to Transcriptomic Heterogeneity in Glioma

**DOI:** 10.3390/cancers18111825

**Published:** 2026-06-02

**Authors:** Syeda Maheen Batool, Hanna Lee, Koushik Muralidharan, Saad Murtaza Khan, Ana K. Escobedo, Denalda Gashi, Kesli Faber, Nina R. Barretts, Emil Ekanayake, Tiffaney Hsia, Yana Al-Inaya, Aishwarya Kosgi, Julie J. Miller, Daniel P. Cahill, Gavin P. Dunn, Bryan D. Choi, Allegra A. Petti, Bob S. Carter, Leonora Balaj

**Affiliations:** 1 Department of Neurosurgery, Massachusetts General Hospital, Harvard Medical School, Boston, MA 02114, USA; smbatool@mgh.harvard.edu (S.M.B.); hlee88@mgh.harvard.edu (H.L.); kmuralidharan@scripps.edu (K.M.); skhan41@mgh.harvard.edu (S.M.K.); akescobedo@mgh.harvard.edu (A.K.E.); gashidenalda@gmail.com (D.G.); kfaber@mgh.harvard.edu (K.F.); nbarretts@mgh.harvard.edu (N.R.B.); emil.ekanayake@gmail.com (E.E.); tiffaney.hsia@umassmed.edu (T.H.); yannaalinaya@gmail.com (Y.A.-I.); akosgi@mgh.harvard.edu (A.K.); cahill@mgh.harvard.edu (D.P.C.); gpdunn@mgh.harvard.edu (G.P.D.); bchoi@mgh.harvard.edu (B.D.C.); apetti@mgh.harvard.edu (A.A.P.); bob.carter@hsc.utah.edu (B.S.C.); 2 Translational Neuro-Oncology Laboratory, Massachusetts General Hospital, Harvard Medical School, Boston, MA 02114, USA; julie.miller@mgh.harvard.edu

**Keywords:** N6-methyladenosine, glioma, IDH1, isoforms, nanopore sequencing, epitranscriptomics

## Abstract

Characterizing the m6A epigenetic landscape is essential for understanding glioma biology, yet transcriptome-wide mapping of these modifications at isoform resolution across specific tumor subtypes has remained limited. Conventional short-read approaches lack the capacity to resolve full-length transcript isoforms or assign m6A modifications to individual transcripts, representing a critical gap in glioma where alternative splicing is pervasive. Methods: We performed direct RNA nanopore sequencing and transcriptome-wide m6A analysis in 14 glioma tumor tissues, including IDH1-mutant astrocytoma, oligodendroglioma, and IDH1 wild-type glioblastoma, enabling isoform-resolved profiling not accessible by conventional short-read approaches. m6A sites were predicted computationally using the m6Anet deep learning framework, which has been independently benchmarked against MeRIP-seq-derived sites, and high-confidence calls were defined at a probability threshold of ≥0.9 and required detection across multiple patients within each subtype. Results: IDH1-mutant gliomas showed a higher overall burden of computationally inferred m6A-modified sites, transcripts, and genes than IDH1 wild-type glioblastoma, along with variation in transcript biotypes, regional distribution of m6A sites, and extent of isoform methylation. Differential methylation analysis identified subtype-specific patterns of m6A localization, many of which were observed without corresponding changes in gene-level expression, indicating that m6A variation represents a post-transcriptional regulatory layer not captured by gene-level analysis alone. Integration of gene expression, isoform usage, and m6A status further identified variation in isoform composition and transcript features between astrocytoma and glioblastoma. Analysis of m6A regulators showed subtype-associated expression patterns among readers, writers, and erasers, and exploratory analyses identified isoform-level associations with survival that were not apparent at the gene level. Conclusions: Overall, these data describe subtype-specific patterns of m6A marking and isoform architecture across glioma tissues, derived from computational inference using direct RNA sequencing in a modestly sized cohort and warrant validation by orthogonal methods in larger studies. These findings are consistent with concurrent independent evidence that isoform-specific m6A deposition is evolutionarily conserved across mammals and that long-read isoform resolution reveals transcript diversity in glioma not captured by gene-level analysis. While cohort size and the absence of orthogonal site-level validation suggest that the data require cautious interpretation, this work provides a hypothesis-generating resource and methodological framework for future mechanistic and translational investigation of the glioma epitranscriptome.

## Simple Summary

Gliomas are heterogeneous brain tumors, yet their subtype-specific RNA methylation patterns and relationship to transcript architecture remain poorly characterized. Conventional approaches to studying RNA modifications, such as methylated RNA immunoprecipitation sequencing, lack the resolution to distinguish modification patterns across individual transcript isoforms, which is a critical limitation in glioma, where alternative splicing and noncoding isoform expression are pervasive. To address this, we applied direct RNA nanopore sequencing to map N6-methyladenosine (m6A) modifications at isoform resolution in 14 patient-derived glioma tissue samples, spanning IDH1-mutant astrocytoma and oligodendroglioma and IDH1 wild-type glioblastoma. We observed that IDH1-mutant gliomas exhibit a higher burden of computationally inferred m6A-modified sites and distinct regional distribution patterns compared to IDH1 wild-type glioblastoma. These differences often occurred without corresponding changes in gene-level expression and were associated with differences in isoform usage and transcript architecture, indicating that m6A represents a post-transcriptional regulatory layer not captured by conventional transcriptomic analysis. The importance of isoform-resolved approaches in glioma is independently supported by concurrent work demonstrating that single-cell long-read sequencing reveals hundreds of cell-population-specific isoforms in GBM that are invisible to short-read methods and that a substantial proportion of evolutionarily conserved m6A sites across mammals display isoform-specific deposition. Despite the modest cohort size and use of computational m6A inference, these data provide a hypothesis-generating, isoform-resolved resource for identifying novel molecular targets and biomarkers stemming from RNA-level modifications in glioma subtypes, and lay the groundwork for future functional and translational studies.

## 1. Introduction

Gliomas are the most common primary brain tumors in adults [1]. Despite significant progress in understanding their molecular landscape, the prognosis for patients with high-grade gliomas, particularly glioblastoma (GBM), remains dismal, with median survival under two years [1,2]. In contrast, glioma patients carrying mutations in isocitrate dehydrogenase 1 (IDH1), are generally associated with a more favorable clinical course [3], although outcomes still vary substantially by tumor type and grade. These clinical differences are reflected in distinct molecular phenotypes and have established IDH1 mutation status as a critical determinant in glioma classification and management [3,4].

A hallmark of IDH1-mutant gliomas is widespread epigenetic reprogramming, driven by accumulation of the oncometabolite D-2-hydroxyglutarate (2-HG), which inhibits α-ketoglutarate (α-KG)-dependent enzymes including DNA and histone demethylases [5]. This leads to global DNA hypermethylation and altered chromatin states that contribute to transcriptional silencing and tumor evolution [5]. While these DNA-level changes are well described, much less is known about how post-transcriptional mechanisms, specifically RNA modifications, shape glioma biology. Among the >170 known RNA modifications, N6-methyladenosine (m6A) is the most abundant internal mark on mRNA and plays essential roles in RNA splicing, translation, stability, and degradation [5,6,7]. The distinct groups of binding proteins, widely known as “m6A regulators” (readers, writers, and erasers) cooperatively interact to deposit, remove, and recognize m6A [8]. The dynamic and reversible changes in m6A influence the key stages of the RNA life cycle including splicing, nuclear export, degradation, and translation [8,9]. Importantly, m6A marks are removed by enzymes that rely on α-KG, such as the “erasers” FTO and ALKBH5, suggesting that IDH1 mutation may also remodel the glioma epitranscriptome [10].

Emerging evidence links m6A to diverse cancer phenotypes including proliferation, therapy resistance, and immune evasion, yet its role in glioma remains incompletely understood. Among the m6A regulators, studies have described METTL3-dependent regulation of RNA processing and stabilization in glioma stem cells (GSCs) with downstream impact on gliomagenesis [11,12]. Prior studies have mostly relied on methylated RNA immunoprecipitation sequencing (MeRIP-seq), which lacks isoform resolution and site specificity [13]. In gliomas, where alternative splicing and noncoding isoform expression are pervasive, this represents a critical limitation [13,14]. Indeed, a recent single-cell long-read RNA sequencing study in GBM demonstrated that conventional short-read approaches fail to resolve full-length isoforms, identifying hundreds of isoforms with differential transcript usage across distinct tumor cell populations, 6524 isoforms absent from existing annotations, and 179 that were tumor-specific [15]. Moreover, the interplay between m6A methylation, transcript structure, immune signaling, and clinical outcomes across glioma subtypes has not been systematically examined in patient-derived tissue using isoform-resolved methods. Long-read RNA sequencing technologies, such as those from Oxford Nanopore Technologies (Oxford, UK), overcome these limitations by providing single-nucleotide resolution for m6A modifications at the isoform level [16]. Additionally, recent advancements in deep learning-based m6A prediction models allow for high-resolution and transcriptome-wide coverage of m6A sites [17]. The importance of isoform-resolved m6A profiling is further underscored by recent evolutionary analyses demonstrating that 27.3% of conserved m6A sites across mammals display isoform-specific deposition, suggesting that modification topology at the transcript level is functionally constrained and cannot be inferred from gene-level summaries alone [18].

In this study, we profile the m6A RNA landscape in IDH1-mutant gliomas (*n* = 8) and IDH1 wild-type glioblastomas (GBM, *n* = 6). Our approach utilized the direct RNA sequencing platform of Oxford Nanopore Technologies, enabling full-length, single-molecule transcript analysis while preserving native RNA modifications. We applied the neural network model m6Anet to predict m6A modifications at a single-nucleotide resolution at the site, transcript, and gene levels, using a high-confidence probability threshold benchmarked against MeRIP-seq-derived sites in its original validation [17]. We then integrated m6A mapping with transcript-region annotation, gene expression, isoform usage, and RNA biotype information, and characterized subtype-associated post-transcriptional regulatory patterns across glioma subtypes. Finally, we evaluated exploratory associations between m6A modifications and clinical outcome measures to assess their potential relevance in glioma. These data provide a hypothesis-generating, isoform-resolved map of the m6A landscape in glioma subtypes, with implications for RNA biology, biomarker discovery, and therapeutic development, and lay the groundwork for future functional and translational studies.

## 2. Materials and Methods

### 2.1. Study Population

The study population (*n* = 14) included patients 18 years or older with histopathologically confirmed IDH1-mutant (*n* = 8, astrocytoma [AA]), *n* = 6; oligodendroglioma [OO], *n* = 2) or IDH1 wild-type glioblastoma (GBM, *n* = 6) who underwent surgery at Massachusetts General Hospital (MGH) for biopsy or resection of a primary brain lesion. Exclusion criteria for the cohort included history of primary or metastatic cancers, active infectious disease (including SARS-CoV-2), and enrollment in clinical trials. All samples were collected with informed consent under the Partners institutional review board (IRB)-approved protocol 2017P001581. The Patient demographics and clinical details are depicted in Supplementary Table S1.

### 2.2. Tumor Tissue Processing

Tumor tissue aliquots were collected during neurosurgical resection or biopsy. The tumor tissue was micro-dissected and suspended in RNAlater (Ambion, Austin, TX, USA) or flash-frozen and stored at −80 °C.

### 2.3. Total RNA Isolation

Frozen tissue was thawed and lysed in 1–2 mL of ice-cold TriZol Reagent (ThermoFisher Scientific, Waltham, MA, USA). The lysate was homogenized by passing through a 20-gauge RNAse-free needle 10 times. The total RNA was then extracted as per the manufacturer’s protocol and eluted in nuclease-free water (Invitrogen, Carlsbad, CA, USA). Both RNA quantity and quality were assessed for purity with a Nanodrop One spectrophotometer (Wilmington, DE, USA). An Agilent RNA 6000 pico kit was used with an Agilent Technologies 2100 Bioanalyzer (Santa Clara, CA, USA) to determine the concentration and RIN (RNA Integrity Number) value of the samples.

### 2.4. Ethanol Precipitation

To remove potential contaminants and carry-over inhibitors, purification via ethanol precipitation was performed at multiple stages of the workflow: post extraction, post demethylation, and post poly(A)+ enrichment. To do this, RNA was combined with 0.1 volume of 3 M, pH 5.2 sodium acetate and 3 volumes of ice-cold, 100% molecular biology-grade ethanol (Sigma-Aldrich, St. Louis, MO, USA). The ethanolic solution was stored at −20 °C overnight. Following this, RNA was recovered by centrifugation at 16,000 *g* for 30 min at 4 °C. The supernatant was carefully aspirated without disturbing the pellet. Subsequently, the pellet was washed with 0.5 mL of ice-cold, freshly prepared 70% ethanol. This was followed by centrifugation at maximum speed for 10 min at 4 °C. The supernatant was removed and the tube was left open at room temperature to ensure that the last traces of fluid had evaporated. The pellet was then dissolved and resuspended in nuclease-free water (Invitrogen, Carlsbad, CA, USA).

### 2.5. Enzymatic Demethylation

The total RNA extracted from each tumor tissue sample was equally split into two aliquots for subsequent demethylation or mock treatment. Given the variability in RNA yield from each tissue sample, up to 200 μg of RNA was either demethylated or mock-treated with the active recombinant FTO/ALKBH5 protein (Abcam, Cambridge, UK) at a 1:0.3 molar ratio in a 500 µL reaction, as previously described by Zheng et al., in 50 mM HEPES (Sigma-Aldrich, St. Louis, MO, USA), 100 µM 2-oxoglutarate (Sigma-Aldrich, St. Louis, MO, USA), 100 µM ascorbate (Sigma-Aldrich, St. Louis, MO, USA), 50 µM Ammonium (II) Iron Sulfate (Sigma-Aldrich, St. Louis, MO, USA), 1 mM TCEP (Sigma-Aldrich, St. Louis, MO, USA), and 50 U of RNAse-Inhibitor (Scientific, Waltham, MA, USA). Care was taken to avoid introduction of RNAses, and all solutions were prepared in nuclease-free water (Ambion, Austin, TX, USA). RNA was ethanol-precipitated and eluted in 100 µL of nuclease-free water.

### 2.6. Poly(A)+ Isolation

Post enzymatic treatment with ALKBH5 or mock treatment, the RNA samples were enriched for poly(A)+ species using the NEBNext^®^ Poly(A) mRNA Magnetic Isolation Module (New England Biolabs, Ipswich, MA, USA), according to manufacturer recommendations. All enrichment reactions were scaled up according to input RNA quantity, using 5 µg as an upper limit for individual samples. Eluted poly(A)+ RNA was then assessed for quality and concentration by the RNA Pico mRNA Assay (Agilent Technologies, Santa Clara, CA, USA).

### 2.7. Library Preparation and Sequencing

Demethylated and mock-treated poly(A)+ RNA from the NEBNext poly(A) isolation module (New England Biolabs, Ipswish, MA, USA) was eluted according to the manufacturer’s instructions and then ethanol-precipitated. RNA was pelleted and resuspended in 10 µL of nuclease-free water (Invitrogen, Carlsbad, CA, USA). A volume of 1 µL was used for the RNA Pico mRNA Assay as a quality check. The remaining 9 µL was used as input for library preparation. Libraries were prepared using the SQK-RNA002 kit (Oxford Nanopore Technologies, Oxford, UK) with selected modifications based on previous optimization runs: the RTA and RMX ligation times were extended to 25 min, the elution times were extended to 15 min, the bead-binding times on the Hula mixer were extended to 7 min, and a Superscript IV (ThermoFisher Scientific, Waltham, MA, USA) was used instead of a Superscript III (ThermoFisher Scientific, Waltham, MA, USA). As such, the thermocycling conditions were modified, and the RTA-adapted RNA was reverse-transcribed at 53 °C for 50 min, with reaction inactivation at 80 °C for 10 min, before holding at 4 °C. Following library preparation, the demethylated and mock-treated poly(A)+ RNA samples were sequenced on a MinION sequencer using R9 flow cells (Oxford Nanopore Technologies, Oxford, UK) for 24 h, or until refuel of the flow cell resulted in a lack of reads. Live basecalling (fast) was used to monitor Q-score (Q filter ≥ 5) and translocation speed for the purposes of refueling.

### 2.8. Statistical Analysis

#### 2.8.1. Raw Sequencing Throughput, QC, Genome Alignment, and Quantification

The total RNA extracted from each patient tissue sample was split into two aliquots, demethylated along with a mock control, and sequenced in parallel. Raw FAST5 files were compiled for each sequencing run, and pass reads (qscore > 7) were basecalled using Dorado (0.5.0 + 0d932c0) using the model rna002_70ps_fast@v3. The resulting FASTQ files were processed using nanoseq [19] (3.1.0) (https://github.com/nf-core/nanoseq/tree/dev; accessed on 22 May 2026), an analysis pipeline for direct RNA sequencing data. It comprises raw read QC, alignment, and quantification.

During the first part of nanoseq, QC metrics from raw reads were generated using Nanoplot. Next, reads were aligned to the human (GRCh38) genome using minimap2 (2.17-r941). Post alignment, SAM files were converted to sorted BAM files using samtools (1.16.1) and mapping metrics were presented using MultiQC (1.11). Finally, nanoseq utilized bambu (3.0.8) to quantify human genome alignments and generate gene counts and normalized abundances. The resulting raw count data were imported into R (version 4.4.0) for downstream analyses.

#### 2.8.2. Quality Control Metrics for ALKBH5-Treated and Untreated Samples

Initially, the study design was based on MasterofPores for paired m6A modification prediction. However, while the analysis was ongoing, m6Anet was developed, which enables accurate prediction of m6A modifications directly from native nanopore signals without the need for a demethylated reference sample. Before combining the conditions, a systematic QC analysis was performed to determine whether enzymatic demethylation had introduced meaningful transcriptomic differences that would confound pooling. PCA of gene expression data showed that treated and untreated libraries from the same patient clustered by patient identity rather than by treatment condition across AA, OO, and GBM cohorts (Supplementary Figure S3a–c). Venn diagram analysis demonstrated that a large majority of the detected genes were shared between conditions within each patient (Supplementary Figure S3d). The distribution of high-confidence m6A sites (probability modified > 0.9) was also highly similar between ALKBH5-treated and untreated RNA samples both globally and at the individual patient level (Supplementary Figure S3e,f), with no consistent directional shift in site counts. On the basis of this evidence that treatment did not meaningfully alter gene expression profiles or m6A detection patterns, the treated and untreated libraries were pooled per patient prior to m6Anet inference, increasing per-patient read depth for modification calling. For differential gene and isoform expression analyses, raw counts were aggregated across conditions per patient prior to DESeq2 analysis, maintaining the patient as the unit of analysis. We note that these QC comparisons do not constitute a formal demethylation efficiency assay, and the degree of demethylation achieved in individual samples was not directly quantified; this is acknowledged as a limitation.

#### 2.8.3. Differential Expression and Isoform Usage Analysis

Differential gene and isoform analysis was performed using DESeq2 [20] (1.45.3) in R. Prior to analysis, for each patient with ALKB-treated and untreated conditions, raw counts were aggregated across conditions. Lowly expressed genes were filtered out by removing genes with less than 10 counts across all samples. The remaining counts were normalized using DESeq’s internal size factor estimation. Log2 fold changes and adjusted *p*-values (Benjamini–Hochberg) were calculated, with an adjusted *p*-value (FDR) threshold of 0.05. Transcripts and genes with a fold change of at least 1.5 (log2fFC > 0.58, log2FC < −0.58) and an adjusted *p*-value less than 0.05 were considered significantly differentially expressed.

Differential isoform usage analysis was performed in R using IsoformSwitchAnalyzeR [21] (2.5.0). The aggregated isoform counts and abundances were input along with the human (GRCh38.p14) annotation and transcriptome files. Single isoform genes were filtered out during the preFilter() step since these genes cannot exhibit changes in isoform usage. Statistical analysis was performed with isoformSwitchTestSatuRn(). We required a difference in isoform proportions between classification groups of >0.2 and an FDR-adjusted *p*-value of <0.05 for significance. The functional consequences of the identified isoform switches were generated using the analyzeSwitchConsequences() function. This analysis identified changes in key functional domains such as coding potential, exon loss, domain loss, and isoform length. Alternative splicing analysis was performed using the extractSplicingEnrichment() function, which identified and assessed the significance of specific events such as alternative 3′ acceptor site (A3) gain or loss.

#### 2.8.4. Transcriptome-Wide m6A Modification Sites

We used m6Anet [17] (2.1.0), a machine learning-based tool, to detect m6A sites in DRACH motifs from our direct RNA reads in all samples. m6Anet provides probabilistic predictions of m6A modification based on nanopore signal features and sequence context and has been benchmarked against MeRIP-seq-derived m6A sites in its original validation (Hendra et al., Nat Methods, 2022) [17]. It should be noted that m6Anet predictions in the present study have not been independently validated by MeRIP-seq, m6A-IP, or site-specific quantitative methods in this cohort, and all site calls should be interpreted as probabilistic inferences. Input data included raw nanopore FAST5 reads, which were aligned to the reference genome using minimap2. After alignment, m6Anet was employed to predict m6A modification sites based on sequence context and nanopore signal patterns. As m6Anet supports pooling over replicates, treated and untreated samples for each patient were pooled prior to the inference step. The output of m6Anet provided predicted m6A sites with probability that the site is modified, the transcript position of the site, the 5-mer motif of the site, and the estimated percentage of reads in the site that is modified. For our analysis, we defined high-confidence m6A sites as those with a probability modified value greater than or equal to 0.9. This threshold was selected to prioritize specificity over sensitivity, consistent with the m6Anet benchmarking data in which higher probability thresholds correspond to increased precision; a lower threshold would increase site yield but come at the cost of reduced specificity. Group-level m6A sites were defined as those detected in at least three patients within the AA and GBM groups, and all detected sites within the OO group.

#### 2.8.5. Distribution of Modified Sites

To analyze the distribution of m6A-modified sites across transcript regions, custom R scripts were used to calculate the lengths of the 5′ UTR, CDS, and 3′ UTR regions. For each transcript, the coding sequence (CDS) length was determined by summing exon lengths, while the UTR lengths were calculated based on the start and end coordinates. Transcripts with missing UTR annotations were assigned a length of zero for the missing regions. To visualize the distribution of m6A-modified sites across these regions, a custom function was implemented in Python (Version 3.12.13).

The transcript position of each modification site was compared with the UTR and CDS regions to determine if the site was located within the 5′ UTR, CDS, or 3′ UTR. The relative position within each region was calculated, and kernel density estimation (KDE) plots were generated to visualize the relative density of m6A sites across these transcript regions. Modifications grouped by transcript biotypes (protein-coding, nonsense-mediated decay, retained-intron) and methylation status were visualized.

#### 2.8.6. Identification of Hyper- and Hypo-m6A-Methylated Transcripts

To identify commonly modified sites within each tumor classification, we first filtered for sites present in at least three patients with AA, at least three patients with GBM, and at least one patient with oligoastrocytoma (OO). For these commonly identified sites within each group, we then identified transcripts containing sites common to all three classifications (AA, GBM, and OO). For these commonly modified transcripts, we calculated the average modification ratio for each site within each classification. Finally, to calculate the weighted modification ratio for these commonly modified transcripts, we summed the modification ratios of all sites within each transcript and divided by the transcript length. This approach ensured that transcripts with more modified sites contributed proportionally while preventing length bias, allowing for a more accurate comparison of modification levels across transcripts of varying lengths. To identify hypermethylated and hypomethylated transcripts, we calculated the log2 fold change of the weighted modification ratio for each pairwise comparison (AA vs. OO, OO vs. GBM, AA vs. GBM). A positive log2FC indicated that a transcript was hypermethylated in AA (vs. GBM), AA (vs. OO), or OO (vs. GBM), while a negative log2FC indicated that a transcript was hypermethylated in GBM (vs. AA), OO (vs. AA), or GBM (vs. OO).

#### 2.8.7. Functional Analysis of Isoform Switching and Hyper- and Hypo-m6A-Methylated Genes

Functional analysis was performed using ClusterProfiler [22] (4.14.6) for all gene ontologies (Biological Process, Molecular Function, and Cellular Component) and KEGG pathways. The enrichGO function was applied for genes that were upregulated and had m6A modifications in each tumor classification.

#### 2.8.8. m6A Regulator Correlation with Survival Outcomes

Clinical metadata and expression data for m6A regulators included overall survival time (in days), vital status (coded as 1 = deceased, 0 = censored), IDH1 status, and normalized transcript expression values. To evaluate the prognostic significance of the individual m6A regulators, univariate Cox proportional hazards models were fit for each gene using the coxph() function from the survival [23] package in R. For each transcript, a model of the form Surv(time, status)~transcript expression was fit to estimate the hazard ratio (HR), 95% confidence interval, and *p*-value. Variables not converging were excluded. Hazard ratios greater than 1 were interpreted as indicating higher risk associated with increased expression, while values less than 1 suggested a better prognosis with increased expression. Transcripts with a *p*-value less than 0.05 were considered statistically significant. A forest plot was generated using ggplot2 to visualize the results, with error bars representing 95% confidence intervals and color coding to indicate statistical significance.

#### 2.8.9. Gene Fusion Detection

Gene fusions were quantified using JAFFA (https://github.com/Oshlack/JAFFA/wiki; accessed on 22 May 2026; version 2.3). FASTQ files were used as input and the JAFFA.groovy script was used with the accurate ONT aligner minimap2 to maximize sensitivity for fusion detection. The output includes genes involved in the fusion event, the position of the fusion, the number of reads, the classification (HighConfidence, MediumConfidence, LowConfidence, and PotentialTransSplicing), and whether or not the fusion is known.

## 3. Results

### 3.1. IDH1 Mutation Is Associated with a Distinct m6A Epitranscriptomic Landscape in Glioma

We performed transcriptome-wide m6A mapping in 14 glioma tumor RNA samples using Nanopore direct RNA sequencing (Supplementary Figures S1–S3; see Section 2). The cohort included IDH1-mutant gliomas (*n* = 8), comprising astrocytoma (AA, *n* = 6) and oligodendroglioma (OO, *n* = 2) and IDH1 wild-type glioblastoma (GBM, *n* = 6). m6A sites were predicted using m6Anet [17] (see Section 2, Supplementary Figures S1c and S3).

**Figure 1:**
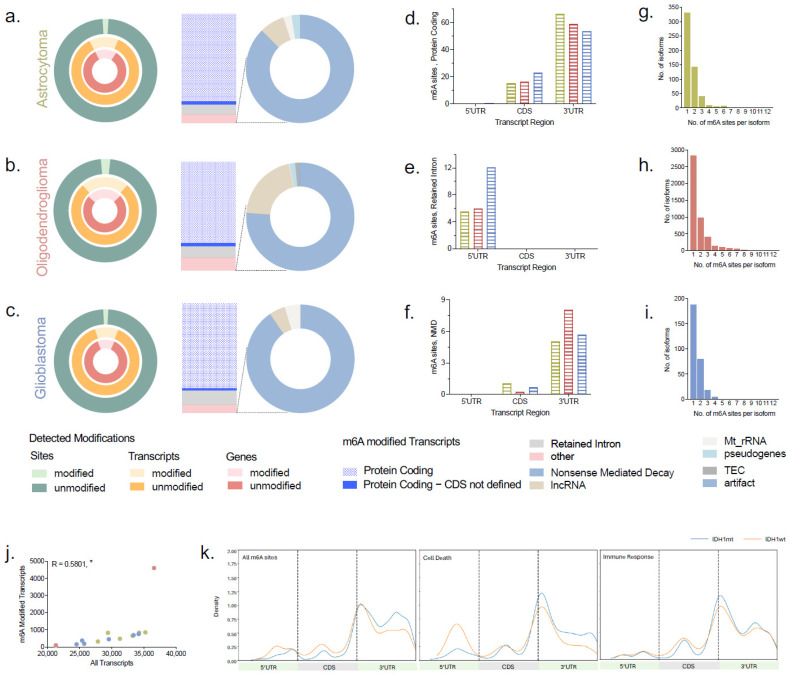
Quantitative and qualitative analysis of m6A-modified sites, transcripts, and genes in the IDH1-mutant vs. wild-type gliomas. (**a**–**c**) Pie charts (**left**) quantifying the prevalence of m6A modifications (probability modified >0.9) out of all detected sites (green), transcripts (orange), and genes (red) within each glioma subtype (astrocytoma, oligodendroglioma, glioblastoma). The identified m6A transcripts in each group are further analyzed to quantify the protein-coding and other m6a RNA biotypes using stacked bars and pie charts (**right**). (**d**–**f**) Bar graphs comparing the number of m6A sites localized along the transcript regions (5′ UTR, CDS, 3′ UTR) across the glioma subtypes (astrocytoma; green, oligodendroglioma; red, glioblastoma; blue) in the common m6A RNA biotypes: (**d**) protein-coding, (**e**) retained-intron, (**f**) nonsense-mediated decay (NMD). (**g**,**h**) Bar graphs quantifying the number of single- (1 m6A site) vs. multi- (>1 m6A site) methylated transcripts in: (**g**) astrocytoma, (**h**) oligodendroglioma, and (**i**) glioblastoma. (**j**) Correlation between the total number of mapped transcripts and the number of high-confidence m6A-modified transcripts in astrocytoma (green), oligodendroglioma (red), and glioblastoma (blue). Correlation coefficients (R) and corresponding significance value are starred. (**k**) Kernel density estimation (KDE) plots showing the regional distribution of m6A sites across transcripts. Distributions are shown for all m6A sites, sites within genes associated with cell death, and sites within genes associated with immune response, comparing IDH1-mutant (blue) and IDH1 wild-type (orange) samples.

Overall, IDH1-mutant gliomas showed a higher burden of m6A modification than IDH1 wild-type GBM, with greater numbers of modified sites, transcripts and genes across AA and OO (Figure 1a–c, left panel). Among all subtypes, OO had the highest percentage of m6A-modified sites (3.0%, *n* = 8139), transcripts (22.9%, *n* = 4611), and genes (26.5%, *n* = 1614) (Figure 1b, left panel). AA showed intermediate levels (sites: 1.9%, *n* = 850; isoforms: 14.1%, *n* = 536; genes: 16.2%, *n* = 181), whereas GBM had the lowest overall burden (sites: 1.6%, *n* = 421; isoforms: 11.4%, *n* = 290; genes: 12.9%, *n* =86) (Figure 1a–c, left panel).

Although the overall RNA biotype composition was broadly similar across subtypes (Supplementary Figure S4), the distribution of m6a-modified biotypes differed substantially (Figure 1a–c, right panels). In all groups, protein-coding transcripts were the predominant modified RNA class. This enrichment was greatest in AA, where 80.2% of modified transcripts were protein-coding, compared with 74.7% in OO and 77.6% in GBM. In contrast, retained-intron transcripts were more frequently modified in GBM (13.1%) than in AA (9.1%) or OO (10.7%). Among noncoding categories, OO showed the highest enrichment of m6A-modified nonsense-mediated decay transcripts (8.7% versus 6.5% in AA and 6.6% in GBM) and lncRNAs (2.3% versus 0.6% in AA and 0.0% in GBM).

We next examined subtype-specific differences in m6A localization across transcript regions for the major modified biotypes, focusing on protein-coding, retained-intron, and nonsense-mediated decay transcripts (Figure 1d–f). In protein-coding transcripts, m6A sites were concentrated primarily in the CDS and 3′ UTR across all subtypes. However, IDH1-mutant gliomas showed relatively greater 3′ UTR enrichment, seen in 66.2% of sites in AA and 59.1% in OO, compared with 53.7% in GBM. By contrast, GBM showed greater CDS localization (23.0%) than AA (15.3%) or OO (16.5%) (Figure 1d). Retained intron transcripts were modified exclusively in the 5′ UTR, with an approximately twofold higher prevalence in GBM (12.1%) than in AA (5.5%) or OO (6.0%) (Figure 1e). For nonsense-mediated decay transcripts, m6A sites were enriched predominantly in the 3′ UTR, followed by the CDS, across all groups. OO showed slightly greater 3′ UTR enrichment (8.1% versus 5.1% in AA and 5.7% in GBM) and lower CDS enrichment (0.23% versus 1.1% in AA and 0.7% in GBM) (Figure 1f).

To further characterize the modified transcript landscape, we assessed read-length distributions, poly(A) tail length, and fusion detection. Read-length analysis suggested subtype-associated differences in transcript representation, whereas poly(A) tail lengths were broadly similar across groups (Supplementary Figure S4b,c). Fusion transcripts were also detected, although only two passed high-confidence filtering criteria (Supplementary Figure S4d).

We then quantified the prevalence of single-site and multi-site methylated isoforms in AA, OO, and GBM (Figure 1g–i). Consistent with the overall increase in m6A burden in IDH1-mutant gliomas, AA and especially OO showed substantially more single-site and multi-site methylated isoforms than GBM. GBM isoforms were predominantly single-site methylated, but the absolute number of single-site methylated isoforms remained much lower in GBM (*n* = 149) than in AA (*n* = 331) or OO (*n* = 2826). Clear differences also emerged in multi-site methylation. AA isoforms carried up to seven m6A sites per transcript, whereas GBM isoforms reached up to only three sites. OO showed the greatest degree of multi-site methylation, with isoforms carrying up to 12 m6A sites (Figure 1g–i).

We next examined the sequence context of high-confidence m6A sites (probability score ≥ 0.9). Both canonical DRACH motifs (GGACT, GAACA, GAACT) and non-DRACH motifs (GCACA, GGACC, AGACT) were detected in glioma tissue (Supplementary Figure S5a). While the overall composition and probability distribution of canonical DRACH motifs were similar across subtypes, the non-DRACH kmer GGACC was enriched in IDH1-mutant gliomas, accounting for 8.6% of motifs in AA and 10.7% in OO, compared with 3.3% in GBM. In addition, motif localization differed by subtype: GGACT and GAACT were more enriched in the 3′ UTR of IDH1-mutant tumors, whereas GBM showed relatively greater 5′ UTR enrichment of GAACT (Supplementary Figure S5a,b).

We further integrated transcript-region information with chromosomal localization of shared m6A targets across glioma subtypes (Supplementary Figure S5c,d). In GBM, 5′ UTR-associated m6A sites were most frequently localized to chromosomes 6 and 9, whereas the CDS and 3′ UTR sites were distributed more broadly across subtypes (Supplementary Figure S5d).

Correlation analysis showed a positive association between the total number of detected isoforms and the number of m6A-modified isoforms (Figure 1j), indicating that m6A site detection likely remains influenced, at least in part, by sequencing-related factors including transcript abundance, isoform complexity, and sequencing depth.

Finally, we examined m6A distributions within functional ontologies related to cell death and immune response (Figure 1k). In both IDH1-mutant and IDH1 wild-type gliomas, immune response-associated m6A peaks were enriched around the stop codon. In contrast, cell death-related transcripts in IDH1 wild-type tumors showed preferential enrichment in the 5′ UTR. Together, these findings support a subtype-specific m6A landscape in glioma that may reflect differences in RNA fate and regulatory function.

### 3.2. Differential m6A Methylation Reveals Subtype-Specific Transcript Localization and Gene Expression Patterns in Glioma

**Figure 2:**
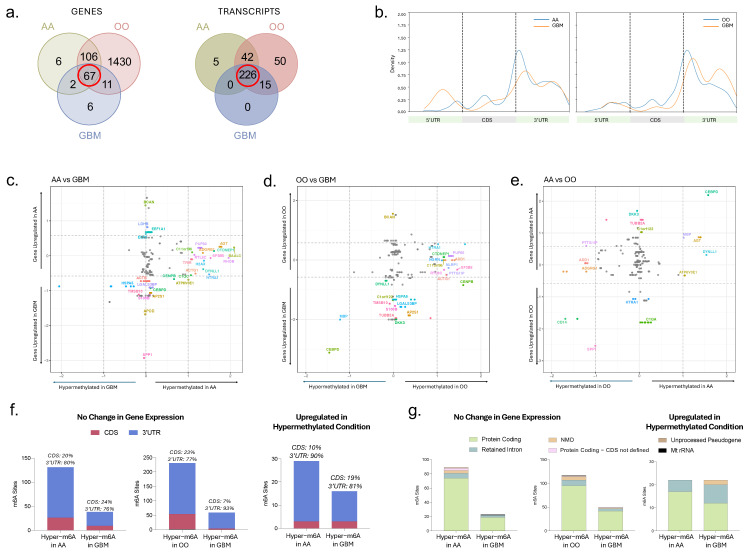
Functional and transcriptomic analysis of hyper- and hypo- m6A-methylated genes. (**a**) Venn diagram depicting the number of unique and shared m6A-modified genes (**left**) and associated transcripts (**right**) across the three glioma subtypes. Common m6A-modified genes and transcripts are circled in red. A total of 226 commonly modified transcripts were used for further analysis. (**b**) KDE plots displaying the regional distributions of hypermethylated transcripts within each condition for astrocytoma vs. GBM (**left**) and oligodendroglioma vs. GBM (**right**). (**c**–**e**) Volcano plots illustrating the differential methylation vs. transcript expression analysis of common m6A-modified genes in glioma subtypes. The *x*-axis represents the log2 fold change (FC) in m6A methylation levels, while the *y*-axis denotes the log2 FC of differentially expressed genes (DEGs). DEGs with Log2 FC > 0.58 or <−0.58 are highlighted in red, while non-significant ones are shown in gray. The analysis is performed across three comparisons: (**c**) astrocytoma vs. GBM, (**d**) oligodendroglioma vs. GBM, and (**e**) astrocytoma vs. oligodendroglioma. (**f**) For genes with no change in expression (**left**) and genes upregulated under hypermethylated conditions (**right**), stacked bar plots show the proportion of m6A-modified sites located in CDS (red) and 3′ UTRs (blue). Comparisons are shown for hypermethylated protein-coding transcripts in astrocytoma (AA) vs. glioblastoma (GBM) and oligodendroglioma (OO) vs. GBM. Percentages indicate the relative distribution of m6A sites within each region. (**g**) For the same gene clusters in f, stacked bar plots show the biotype distribution of hypermethylated transcripts in AA vs. GBM and OO vs. GBM.

To examine the relationship between gene expression and m6A modification, we analyzed differential methylation across 226 transcripts corresponding to 67 genes commonly modified in all three glioma subtypes (AA, OO, and GBM; Figure 2a). IDH1-mutant gliomas (AA and OO) showed a greater number of multi-site methylated transcripts than GBM (Supplementary Figure S6a,b), whereas the number of modified sites per gene was similar across groups (Supplementary Figure S6c,d). A modest positive correlation was also observed between transcript length, number of m6A sites, and modification ratio for transcripts up to approximately 2500 bp (Supplementary Figure S6e,f).

To estimate transcript-level methylation, we summed site-specific modification ratios for each isoform and normalized by transcript length to generate a weighted modification ratio (Section 2; Supplementary Figure S6g). Log2 fold changes in the weighted modification ratio were then calculated for pairwise comparisons between AA and GBM, OO and GBM, and AA and OO to identify relatively hyper- and hypomethylated transcripts.

Analysis of transcript-region localization in hypermethylated transcripts showed subtype-specific distribution patterns (Figure 2b). In AA versus GBM, hypermethylated transcripts in GBM were more frequently localized to the 5′ UTR, whereas those in AA showed greater representation in the CDS and near the stop codon (Figure 2b, left panel). In OO versus GBM, hypermethylated transcripts in OO were more enriched near the stop codon, while those in GBM were shifted toward the distal 3′ UTR (Figure 2b, right panel).

We next compared differential methylation with gene expression status (Figure 2c–g) and found that many methylation differences were observed in the absence of corresponding gene-level expression changes. Specifically, 49, 53, and 59 genes were hypermethylated without differential expression in AA versus GBM, OO versus GBM, and AA versus OO, respectively (Figure 2c–e). These data indicate that differential methylation and differential gene expression were not uniformly coupled across comparisons.

At the same time, hypermethylation was also observed among genes with altered expression and across multiple RNA biotypes, including protein-coding and retained-intron transcripts. In some cases, these patterns were present among distinct isoforms from the same gene (Figure 2f,g; Supplementary Figure S6h).

To further examine these relationships, we assessed region-specific m6A localization in hypermethylated protein-coding transcripts after stratifying genes by expression status (Figure 2f; Supplementary Figure S6h). In AA versus GBM, genes without differential expression showed broadly similar m6A distributions, with a modest increase in 3′ UTR localization in AA compared with GBM (80% versus 76%). Among upregulated genes, hypermethylated transcripts in AA showed greater 3′ UTR representation (90% versus 81% in GBM), whereas GBM showed a relatively larger CDS fraction. In OO versus GBM, genes without differential expression showed a greater proportion of CDS methylation in OO than in GBM (23% versus 7%), with a corresponding reduction in 3′ UTR localization (Figure 2f).

Overall, these analyses describe subtype-specific differences in the regional distribution of m6A methylation and show that these patterns may occur either with or without accompanying differences in gene expression.

### 3.3. m6A-Modified Upregulated Genes Are Associated with Distinct Functional Annotations in IDH1-Mutant and Wild-Type Gliomas

**Figure 3:**
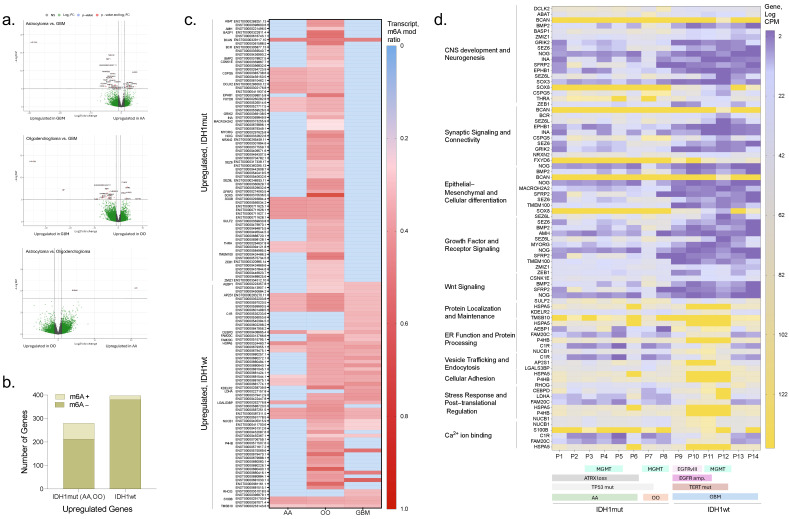
Functional enrichment of m6a-modified upregulated genes in IDH1-mutant and wild-type gliomas. (**a**) Volcano plots showing differentially expressed genes (DEGs) from each pairwise comparison: AA vs. GBM (**top**), OO vs. GBM (**middle**), and AA vs. OO (**bottom**). Significant DEGs (Log2 FC > 0.58 or <−0.58) are highlighted in red. (**b**) Stacked plot quantifying the number of upregulated genes in IDH1-mutant (mut) and IDH1 wild-type (wt) gliomas that are also m6A-modified (light green). (**c**) Heatmap displaying the weighted m6A modification ratio per isoform in upregulated, m6A-modified genes from the mutant (*n* = 68) and wild-type (*n* = 17) group. (**d**) Heatmap of top enriched gene ontology (GO) terms among m6A-modified upregulated genes. The *y*-axis denotes the enriched functional pathway, and the associated genes identified in each ontology term. Pathway enrichment is plotted by patient (rows) and associated gene expression (counts per million, columns), stratified by IDH1 status and key molecular features (EGFR, TERT, ATRX, TP53, MGMT).

We next performed differential gene expression analysis across three comparison groups: (i) AA versus GBM, (ii) OO versus GBM, and (iii) AA versus OO (Figure 3a; see Section 2). Across the comparisons involving IDH1-mutant gliomas and GBM, 212 genes were upregulated in the IDH1-mutant groups (AA versus GBM and OO versus GBM combined), whereas 397 genes were upregulated in GBM (Figure 3a).

To further examine the overlap between gene expression and m6A modification, we filtered the upregulated genes in each comparison to include those with predicted m6A modification in at least one associated isoform (Figure 3b). Among the 212 genes upregulated in the IDH1-mutant group, 68 also contained at least one m6A-modified isoform in either the astrocytoma or the oligodendroglioma condition. In contrast, among the 397 genes upregulated in GBM, 17 contained at least one m6A-modified isoform (Figure 3b).

We then quantified the weighted m6A modification ratio at the isoform level (see Section 2; Figure 3c). Among the 17 genes that were both upregulated and m6A-modified in GBM, two genes, C1R and RHOG, showed modification patterns unique to GBM (Figure 3c). In contrast, many of the upregulated genes in the IDH1-mutant group showed m6A modification patterns that were not observed in GBM, with the greatest number seen in OO. Among genes upregulated in GBM that were also modified across both IDH1-mutant and IDH1 wild-type gliomas, both relatively higher and lower methylated isoforms were observed in GBM compared with AA and OO (Figure 3c).

We next examined the functional annotations of the m6A-modified upregulated genes in the IDH1-mutant and IDH1 wild-type groups using ontology analysis (see Section 2; Figure 3d). The enriched terms differed between the groups. In IDH1-mutant gliomas, enriched categories included CNS development and neurogenesis, synaptic signaling, cellular differentiation, and growth factor and Wnt signaling. In GBM, enriched categories included protein localization and maintenance, endoplasmic reticulum function and protein processing, vesicle trafficking, cellular adhesion, stress response, and Ca2+ ion binding (Figure 3d).

### 3.4. Differential Isoform Usage and m6A-Associated Isoform Patterns in Astrocytoma and Glioblastoma

Differential isoform usage across RNA biotypes was examined in the glioma cohort using an integrated analysis of differential gene expression (DEG), differential isoform expression (DEI), and differential isoform usage (DIU) based on DESeq2 and IsoformSwitchAnalyzeR (see Section 2).

**Figure 4:**
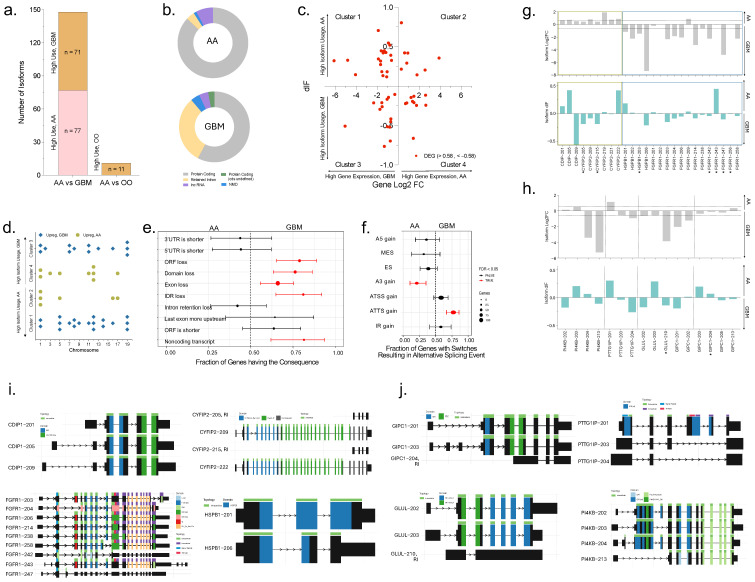
Differential isoform usage between astrocytoma (AA) and glioblastoma (GBM). (**a**) Stacked bar plot quantifying the number of high-usage isoforms in AA vs. GBM and AA vs. OO. (**b**) Pie charts depicting the RNA biotype distribution among high-usage isoforms in AA (*n* = 77, top panel) and GBM (*n* = 71, bottom panel). (**c**) Scatter plots showing the relationship between difference in isoform usage (difference in isoform fraction; dIF, *y*-axis) and gene-level log2 fold change (*x*-axis) in AA vs. GBM. Significant DEGs are highlighted in red. (**d**) Chromosomal distribution of upregulated genes in GBM (blue) and AA (green) linked to high or low isoform usage. (**e**) Consequence enrichment analysis identifying predicted structural consequences of isoform switches between AA and GBM. *Y*-axis: consequence, *x*-axis: fraction of genes having that consequence (95% confidence interval, CI). Dot size correlates with the number of genes with that consequence. (**f**) Alternative splicing event enrichment analysis in AA vs. GBM. (**g**) Differential isoform expression (top, Log2 FC, top panel; gray) and usage (dIF, bottom panel; blue) in selected non-m6A-modified upregulated genes in AA or GBM. (**h**) Differential isoform expression of m6A-modified transcripts with significant dIF usage in AA or GBM. Dotted line indicates a threshold for significance, and isoforms with significant differential usage are starred. (**i**,**j**) Isoform plots of genes depicted in (**g**,**h**).

Subtype comparison identified 159 statistically significant isoform switches across 139 genes, including 148 between AA and GBM and 11 between AA and OO (Figure 4a; Supplementary Figure S7a). Among the 148 isoforms with significant differential isoform usage between AA and GBM, high-usage isoforms were similarly distributed between AA (*n* = 77) and GBM (*n* = 71) (Figure 4a). However, the biotype composition of these isoforms differed between the two groups (Figure 4b). AA contained more high-usage protein-coding isoforms (*n* = 67) than GBM (*n* = 41), whereas retained-intron isoforms were more frequent among high-usage isoforms in GBM (*n* = 22 versus *n* = 3 in AA). lncRNA isoforms were observed at lower numbers in both groups (AA: *n* = 6; GBM: *n* = 3) (Figure 4b).

Joint analysis of differential isoform usage and gene-level expression identified four expression-usage categories (Figure 4c): cluster 1, higher isoform usage in AA with higher gene expression in GBM (*n* = 20); cluster 2, higher isoform usage and higher gene expression in AA (*n* = 6); cluster 3, higher isoform usage and higher gene expression in GBM (*n* = 13); and cluster 4, higher isoform usage in GBM with higher gene expression in AA (*n* = 10) (Supplementary Table S2). In the AA versus OO comparison, 11 genes showed significant isoform switches, and in all cases the switched isoforms were more highly used in OO (Supplementary Figure S7a).

The genes assigned to these clusters showed different chromosomal distributions (Figure 4d). Genes with higher expression in AA (clusters 2 and 4) were more frequently localized to shorter chromosomes, whereas genes with higher expression in GBM (clusters 1 and 3) were distributed more broadly across the chromosomes (Figure 4d).

We next examined structural features associated with isoform switching in AA and GBM using consequence enrichment analysis in IsoformSwitchAnalyzeR (see Section 2). High-usage isoforms in GBM were enriched for several structural features, including complete open reading frame loss, domain loss, exon loss, intrinsically disordered region loss, and noncoding transcripts (Figure 4e). Analysis of alternative splicing events further showed that high-usage isoforms in GBM more frequently included gain of alternative transcription termination sites and loss of alternative 3′ acceptor sites (Figure 4f).

Selected genes illustrated the range of observed isoform usage and gene expression patterns. In AA, higher usage of the protein-coding isoforms CDIP1-205 and CYFIP2-222 was observed together with higher gene expression relative to GBM (Figure 4g,i). Higher usage of the FGFR1-243 nonsense-mediated decay isoform in AA was observed in the setting of lower overall gene expression relative to GBM (Figure 4g,i). In GBM, higher usage of the protein-coding isoform HSPB1-206 was observed together with higher gene expression relative to AA (Figure 4g,i).

We also examined a subset of isoform-switched genes with m6A-modified isoforms, including GIPC1, GLUL, PTTG1IP, and PI4KB. These genes showed differences in isoform usage and expression between groups, with m6A-modified isoforms present among the switched transcripts (Figure 4h,j).

To further describe the genes with isoform switching, we performed gene ontology analysis (see Section 2; Supplementary Figure S7b,c). In AA, the most enriched categories by fold enrichment included p53-mediated intrinsic apoptosis, TNF signaling, protein geranylgeranylation, tyrosine kinase signaling, and guanylyl cyclase signaling. In GBM, enriched categories included oxygen carrier activity, inorganic phosphate transmembrane transport, protein kinase C inhibitor activity, angiogenesis, hydrogen peroxide metabolism, and nitric oxide transport. Categories reduced in AA included inflammation, coagulation, epithelial–mesenchymal transition, and complement, whereas categories reduced in GBM included aldehyde-lyase activity, serine binding, postsynaptic calcium ion regulation, ubiquitin ligase activity, and hydroxymethyl-formyl-transferase activity (Supplementary Figure S7b,c).

In addition to mapping m6A modifications and isoform usage, we examined the functional composition of transcripts detected within each protein-coding gene across glioma subtypes. Since isoform switching can produce transcripts that lose protein-coding capacity, we categorized isoforms within each gene as functional or non-functional. Functional isoforms were defined as protein-coding transcripts, whereas non-functional isoforms included retained-intron (RI), nonsense-mediated decay (NMD), and other noncoding biotypes. Protein-coding isoforms were the predominant class in all subtypes, but their relative abundance differed modestly, accounting for 71% of transcripts in astrocytoma (AA), 70% in oligodendroglioma (OO), and 67% in glioblastoma (GBM) (Supplementary Figure S8a,b).

When examined at the gene level, only 8% to 16% of expressed protein-coding genes were composed predominantly (≥75%) of functional isoforms, indicating that non-functional isoforms contributed substantially to the overall transcript landscape in all glioma subtypes (Supplementary Figure S8a,b).

Several highly expressed genes also showed a substantial proportion of non-functional isoforms in subsets of patients (Supplementary Figure S8a). For example, BCAN showed more than 70% noncoding isoforms in some OO and GBM samples, whereas some AA samples showed substantially lower proportions, in some cases near 5%. HSPA5, which was upregulated in GBM, also showed more than 80% non-functional isoform expression in multiple samples. These patterns illustrate variation in isoform composition across patients and subtypes.

By contrast, genes such as IDH1, MGMT, and BRAF were represented predominantly by protein-coding isoforms across subtypes (Supplementary Figure S8a). Overall, these analyses show that gene-level expression and isoform-level composition do not always align and that a substantial fraction of expressed transcripts in glioma consists of non-functional isoforms.

### 3.5. m6A Regulators and Isoform-Level Associations Across Glioma Subtypes and Clinical Outcomes

**Figure 5:**
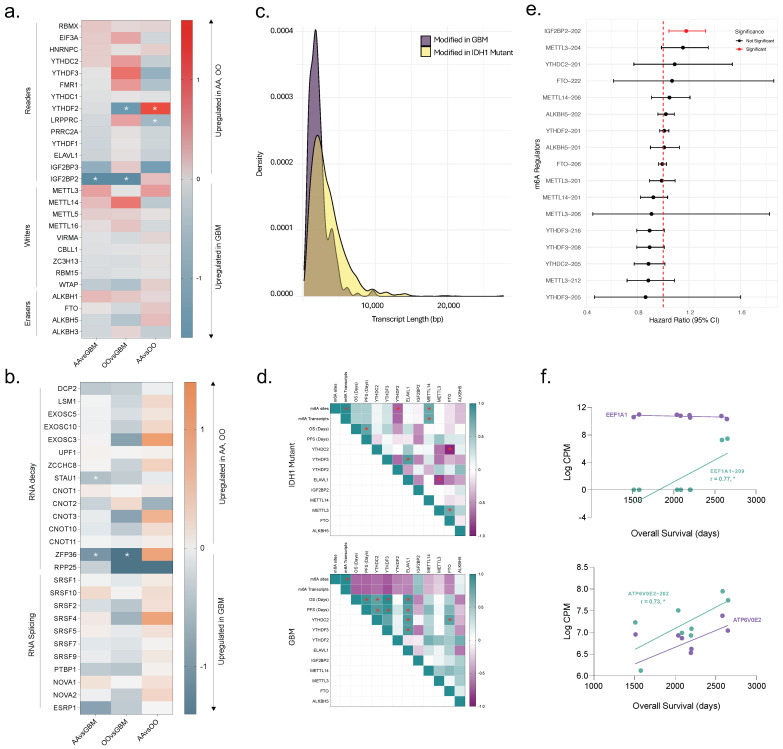
m6A regulators and transcriptomics consequences across glioma subtypes. (**a**) Heatmap of differentially expressed m6A regulators (readers, writers, erasers) across three pairwise comparisons: AA vs. GBM, OO vs. GBM, and AA vs. OO. Genes with significant differential gene expression (log2FC > 0.58, log2FC < −0.58, *p*-value < 0.05) are starred. (**b**) Heatmap of differentially expressed RNA splicing and decay genes across three pairwise comparisons: AA vs. GBM, OO vs. GBM, and AA vs. OO. Genes with significant differential gene expression (log2FC > 0.58, log2FC < −0.58, *p*-value < 0.05) are starred. (**c**) Density distribution comparing transcript lengths of m6A-modified RNAs in IDH1-mutant (yellow) and wild-type (purple) gliomas. (**d**) Correlation matrices of m6A regulator expression, m6A site/transcript abundance, and clinical outcomes (OS, PFS) for IDH1-mutant (**top**) and IDH1 wild-type (**bottom**) patients. Significant correlations (*p* < 0.05) are starred. (**e**) Forest plot of hazard ratios (HR, COX regression analysis) and 95% confidence intervals (CIs) for isoforms of m6A regulators. Significant associations (*p* < 0.05) are highlighted in red. (**f**) Correlation plots showing the association between hypermethylated transcripts and their corresponding gene expression in IDH1-mutant gliomas, as well as overall survival in IDH1-mutant patients. For significant correlations, corresponding correlation coefficients (Rs) and label along with stars for *p*-value significance.

To examine the expression of upstream m6A regulators across glioma subtypes, we analyzed 26 m6A-associated proteins classified as readers, writers, and erasers in AA versus GBM, OO versus GBM, and AA versus OO (Figure 5a). METTL3 and METTL14 showed higher expression in the IDH1-mutant group, whereas ALKBH5 and FTO were higher in the IDH1 wild-type group, although these differences were not statistically significant (Figure 5a). Among reader proteins, YTHDF3 and YTHDC2 were higher in IDH1-mutant gliomas, while YTHDF2 and IGF2BP2 were significantly upregulated in GBM (Figure 5a).

We next examined genes involved in RNA decay pathways (Figure 5b). Several RNA decay-associated factors showed higher expression in GBM, with STAU1 and ZFP36 reaching statistical significance relative to AA and OO (Figure 5b). Analysis of m6A-modified transcript length distributions also showed a significantly greater relative abundance of shorter transcripts in GBM compared with IDH1-mutant gliomas (Figure 5c).

We then assessed whether m6A abundance and regulator expression were associated with clinical outcome measures (Figure 5d). Correlation analyses stratified by IDH status showed modest positive associations between m6A site or the number of m6A-marked transcripts and both overall survival (OS) and progression-free survival (PFS) in the IDH1-mutant group (Figure 5d). In IDH1-mutant tumors, METTL14 expression was positively correlated with m6A site and transcript abundance (r = 0.77), with similar trends observed for METTL3 and FTO (r = 0.75). Negative correlations were observed between YTHDF2 expression and m6A site abundance (r = −0.71), and between FTO and YTHDC2 expression (r = −0.93). In GBM, FTO and YTHDC2 expression were positively correlated (r = 0.82). In the same group, readers’ YTHDC2, YTHDF3, ELAVL1 expressions were positively associated with OS (YTHDC2 r = 0.82, YTHDF3 r = 0.99, ELAVL1 r = 0.91) and PFS (YTHDC2 r = 0.86, YTHDF3 r = 0.95, ELAVL1 r = 0.84).

To identify individual isoforms associated with survival, we performed Cox proportional hazards regression analysis on the corresponding transcripts (Figure 5e). Among the isoforms tested, IGF2BP2-202 showed a statistically significant association with shorter overall survival (HR > 1, *p* < 0.05) (Figure 5e).

We also examined whether transcript-specific m6A modification levels were associated with survival outcomes (Figure 5f). EEF1A1-209, which showed higher methylation in IDH1-mutant tumors, was positively associated with survival in this subgroup (r = 0.77, *p* < 0.05), whereas no significant association was observed at the gene level (*p* = 0.29). Similarly, ATP6V0E2-202 showed higher methylation in IDH1-mutant tumors and a positive association with overall survival (r = 0.73, *p* < 0.05), while no significant gene-level association was detected (*p* = 0.11). These analyses describe isoform-level associations that were not apparent in the corresponding gene-level summaries.

## 4. Discussion

In this study, we applied direct RNA nanopore sequencing for simultaneous, isoform-resolved characterization of m6A sites, transcript biotypes, isoform usage, and transcript architecture across IDH1-stratified glioma subtypes in patient-derived tissue, a resolution not accessible by MeRIP-seq or conventional short-read approaches. Several features of the study distinguish it from prior work. First, isoform-resolved m6A profiling enabled simultaneous characterization of modification sites, transcript biotypes, isoform usage, and transcript architecture within the same sample. Second, the systematic IDH1-stratified design spanning astrocytoma, oligodendroglioma, and glioblastoma provides a subtype-resolved map of the m6A landscape, regional distribution, and isoform composition not previously reported in patient-derived tissue using long-read methods. Third, a substantial proportion of subtype-associated m6A differences were observed in the absence of corresponding changes in steady-state gene expression, revealing that isoform-level RNA modification represents a regulatory layer not captured by conventional transcriptomic analysis. Fourth, the integrative framework combining m6A mapping with differential isoform usage, RNA decay factor expression, and exploratory survival analyses provides a more comprehensive view of post-transcriptional regulation in glioma than previously available from short-read data.

The higher m6A burden observed in IDH1-mutant gliomas in our cohort is broadly consistent with prior glioma studies linking IDH mutation to increased RNA methylation. Pianka et al. reported that IDH1-mutant glioma models and patient tumors show increased m6A in association with FTO inhibition [13], and Steponaitis et al. likewise found higher m6A abundance in low-grade gliomas than in GBM using direct RNA long-read sequencing [24]. Although prior studies largely focused on overall methylation burden, our analysis reveals transcript-level distinctions: IDH1-mutant samples demonstrate increased 3′ UTR enrichment in protein-coding transcripts and a higher prevalence of multi-site methylated isoforms, whereas GBM samples exhibit a larger CDS fraction and enhanced representation of retained-intron methylation. This difference between global burden and transcript-level distribution is also compatible with the observation by Steponaitis et al. that regulator mRNA levels do not necessarily parallel overall m6A abundance in glioma [24].

Another notable finding was that many subtype-associated differences in m6A methylation were observed without corresponding changes in gene-level expression. Across comparisons, a substantial number of hypermethylated genes were not differentially expressed, indicating that variation in m6A marking was not uniformly reflected in steady-state transcript abundance. Differences in the regional distribution of m6A within hypermethylated transcripts were also observed across subtypes, including shifts in 3′ UTR and CDS localization. This partial separation between m6A status and transcript abundance is in-keeping with prior glioma work showing that m6A can be linked to nonsense-mediated decay [25], alternative splicing [26], and transcript turnover [27,28] rather than to a uniform increase or decrease in total RNA output alone.

At the isoform level, we identified widespread switching events, with 148 isoform switches associated with altered expression across 52 genes, consistent with prior reports of splicing deregulation in high-grade gliomas [14,29]. GBM showed greater representation of noncoding isoforms, including retained-intron and nonsense-mediated decay transcripts, several of which also carried m6A marks in the 5′ UTR. These transcripts were frequently associated with shortened UTRs, loss of open reading frames, exon loss, and loss of functional domains or intrinsically disordered regions, features that may influence transcript stability, translation, or protein output. Considered together with the higher expression of splicing (SRSF genes) and decay-related factors (STAU1 and ZFP36) in GBM, these observations further suggest that aggressive gliomas differ not only in gene expression, but also in isoform usage, transcript structure, and half-life. The extent of isoform diversity in GBM is further highlighted by a recent single-cell long-read RNA sequencing study identifying hundreds of isoforms with differential transcript usage across distinct tumor cell populations, including 6524 isoforms absent from existing annotations and 179 that were tumor-specific [15], underscoring that bulk long-read profiling as performed here captures only a population-level average of what is likely a more complex, cell-type-specific isoform landscape.

We also observed subtype-specific differences in the sequence context of m6A sites. Both canonical DRACH and non-DRACH motifs were detected across subtypes, with the non-DRACH kmer GGACC enriched in IDH1-mutant gliomas relative to GBM (8.6% in AA, 10.7% in OO versus 3.3% in GBM; Supplementary Figure S5a). The biological relevance of non-DRACH m6A sites is supported by a recent cross-species evolutionary analysis demonstrating that 21.7% of evolutionarily conserved m6A sites across mammals use non-canonical motifs, and that these sites display higher modification stoichiometry than canonical DRACH sites and stronger evolutionary sequence conservation as measured by phyloP scores [18]. While this does not validate our specific non-DRACH calls, it indicates that non-DRACH m6A deposition is a genuine biological phenomenon rather than an artifact of nanopore detection and strengthens the rationale for including these sites in descriptive analyses pending site-specific experimental verification.

Subtype-specific differences were also evident in the genes and isoforms associated with m6A modification. Among upregulated genes, IDH1-mutant gliomas contained a larger number with m6A-modified isoforms than GBM, and the functional annotations of these genes differed between groups, with IDH1-mutant tumors enriched for categories related to neurogenesis, synaptic signaling, and differentiation, and GBM enriched for protein processing, vesicle trafficking, adhesion, and stress-response pathways. At the isoform level, AA and GBM also differed in isoform usage, biotype composition, and structural features of isoform switching, with AA showing a greater number of highly used protein-coding isoforms and GBM showing greater representation of retained-intron isoforms and switches associated with loss of coding features. These observations are in line with prior GBM studies indicating that m6A machinery can intersect with transcript processing pathways, including splicing and nonsense-mediated decay [11,25].

We next examined subtype-specific patterns in m6A regulators, RNA decay factors, and isoform-level associations with outcomes. Differences in reader protein expression were accompanied by differences in RNA decay and RNA processing factors across glioma subtypes. In GBM, higher YTHDF2 expression was observed together with increased expression of several decay-associated factors, whereas IDH1-mutant gliomas showed higher YTHDF3 expression along with proteins linked to RNA stabilization and translation. METTL3 and METTL14 tended to be higher in IDH1-mutant tumors, whereas ALKBH5 and FTO tended to be higher in GBM, although these differences were not statistically significant in our cohort. ALKBH5 has been previously linked to GBM stem-like cell maintenance, and METTL3 has been associated with more aggressive behavior in cancer [30] as previously reported across large glioma cohorts [31], supporting the idea that regulator expression differs across glioma states, although reported patterns are not always uniform across these studies likely due to the multifaceted role m6A plays in RNA biology. These regulator expression differences offer a plausible mechanistic context for the m6A topology differences we observe. The greater 3′ UTR enrichment in IDH1-mutant tumors, occurring in a setting of higher YTHDF3 and lower YTHDF2 expression, is consistent with a regulatory environment favoring transcript stabilization or translational enhancement rather than decay. Conversely, the higher YTHDF2 expression and elevated RNA decay factor levels in GBM, together with greater CDS methylation and a higher proportion of shorter m6A-modified transcripts (Figure 5c), point toward a program favoring transcript turnover. These interpretations remain speculative in the absence of direct proteomic or RNA stability data and resolving them will require ribosome profiling or RNA decay assays in matched tumor models. Mechanistic support for these patterns is provided by our companion cell line study (Batool, Lee, Escobedo et al., Biochemistry and Biophysics Reports, 2026, 102614) [26], in which ALKBH5 knockdown in Gli36 glioma cells, modeling the lower ALKBH5 state of IDH1-mutant tumors, redistributed m6A from the CDS toward the 3′ UTR with consequent gene upregulation, while ALKBH5 and IGF2BP2 knockdown each increased retained-intron isoform usage, paralleling the retained-intron patterns observed in GBM. In our data, selected isoform-level associations with survival were observed even when corresponding gene-level associations were not, which further supports the value of transcript-level analysis in glioma tissue. The association between higher IGF2BP2-202 expression and shorter overall survival is directionally consistent with TCGA-based analyses reporting that elevated IGF2BP2 correlates with worse prognosis in GBM, and with the broad transcriptomic shifts observed upon IGF2BP2 knockdown in the cell line study [26]. These survival findings are exploratory and require prospective validation in adequately powered cohorts before m6A isoform signatures can be considered clinically validated biomarkers.

This study has several limitations that should be considered when interpreting the findings. The cohort size was modest (*n* = 14), particularly for the oligodendroglioma subgroup (*n* = 2), limiting statistical power for OO-involving comparisons; replication in larger, prospectively collected cohorts is necessary before any findings can be considered established. The analyses were performed in bulk tumor tissue, where differences in cellular composition between IDH1-mutant and GBM samples, including greater myeloid infiltration and mesenchymal stromal content in GBM, may contribute to observed differences in isoform abundance, RNA decay factor expression, and m6A regulator levels independently of intrinsic tumor cell biology. Cell-type deconvolution was not applied, and single-cell or spatially resolved approaches will be required to disentangle these contributions; the feasibility of single-cell long-read isoform profiling in GBM has recently been demonstrated [15], and applying this approach with concurrent m6A profiling represents a logical next step. m6A sites were predicted computationally using m6Anet rather than validated by orthogonal biochemical methods in this cohort, and all site calls should be interpreted as probabilistic inferences; sequencing depth was not formally equalized across samples, and run-to-run technical variation was not explicitly modeled, though per-run QC metrics were broadly comparable. The survival analyses are exploratory and should not be interpreted as establishing validated clinical biomarkers, and the functional consequences of the observed m6A and isoform switching differences for protein output and cellular behavior remain to be determined experimentally. While the companion cell line study [26] provides mechanistic support for regulator-driven isoform changes consistent with those described here, direct validation of specific isoform switches identified in patient tissue remains a priority for future work.

## 5. Conclusions

Using direct RNA nanopore sequencing we have characterized the m6A landscape across glioma subtypes at the isoform level. Our results indicate that IDH1-mutant and wild-type tumors differ in their computationally inferred m6A marking and transcript features, often without corresponding changes in total gene abundance, suggesting that RNA modification represents a post-transcriptional regulatory layer not captured by conventional gene-level transcriptomic analysis. While the modest cohort size and use of computational m6A inference rather than orthogonal biochemical validation necessitate cautious interpretation, these data provide a hypothesis-generating, isoform-resolved resource for the field. Future work should prioritize independent replication in larger cohorts, orthogonal validation of specific m6A sites, functional experiments targeting individual m6A regulators in glioma models to validate the observed isoform switches, and integration with single-cell and proteomic approaches to resolve the cell-type-specific contributions and downstream functional consequences of these epitranscriptomic differences.

## Data Availability

The sequencing and m6A modification data generated in this study are provided in the Supplementary Information and Source Data file. The raw nanopore sequencing data and m6Anet output are not publicly available but will be available upon request from the corresponding author. Source data are provided with this paper.

## Supplementary Materials

The following supporting information can be downloaded at https://www.mdpi.com/article/10.3390/cancers18111825/s1, Figure S1: Overview of direct RNA sequencing and m6A analysis pipeline; Figure S2: Comparison of sequencing output in ALKBH5-treated and untreated RNA; Figure S3: Comparison of gene expression and m6A site detection with ALKBH5 treatment; Figure S4: Distributions of key transcriptomic features across glioma subtypes; Figure S5: Transcriptomic analysis of detected m6A-modified sites; Figure S6: Commonly modified transcripts across the glioma subtypes demonstrate variation in m6A levels; Figure S7: Isoform switching analysis with gene expression and functional pathways; Figure S8: Analysis of function and non-functional transcript composition among protein-coding genes; Table S1: Clinical characteristics of the patient cohort; Table S2: Gene lists for isoform switching analysis clusters in astrocytoma (AA) versus glioblastoma (GBM).
