# Supplementary material for: IDH1-Associated m6A Methylation Is Linked to Transcriptomic Heterogeneity in Glioma
Source: Cancers (Basel). 2026 Jun 2;18(11):1825. doi: 10.3390/cancers18111825 (PMC13256496; doi:10.3390/cancers18111825)
Supplement: Supplementary file 1 [file cancers-18-01825-s001.zip › cancers-4318059-supplementary.pdf]

Supplementary Figure S1

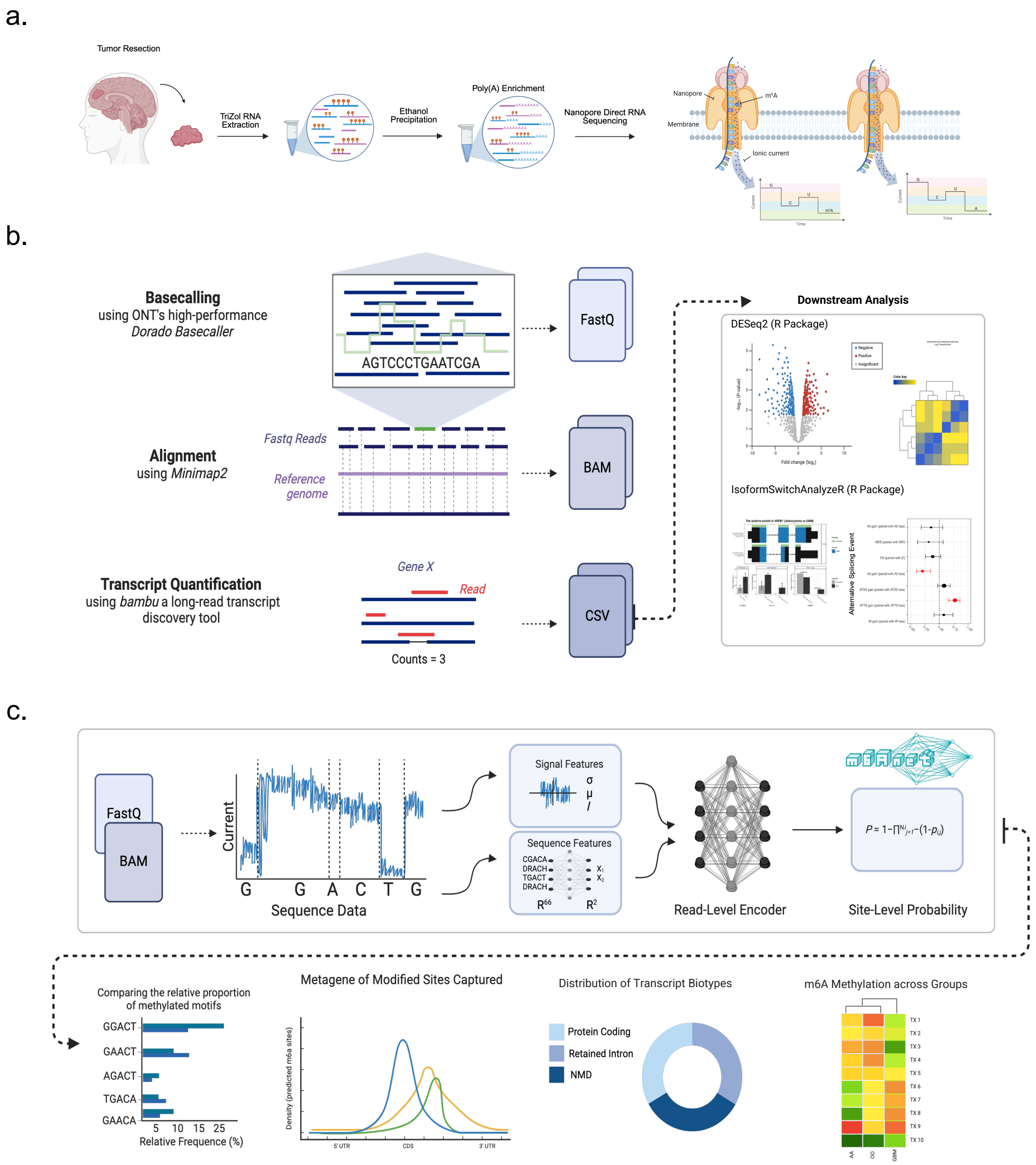

Supplementary Figure S1. Overview of direct RNA sequencing and m6A analysis pipeline. (a) Experimental Workflow. Total RNA from glioma tissue was poly(A) enriched and sequenced using Oxford Nanopore direct RNA sequencing. (b) Post-sequencing analysis. Reads were base-called (Dorado, aligned to the human genome (Minimap2), and quantified using a long-read transcript tool (Bambu). Resulting data (FastQ, BAM, CSV) were used for differential gene and transcript expression (DESeq2) and isoform usage analysis (IsoformSwitchAnalyzeR). (c) m6A modification analysis. Using m6anet, signal and sequence features were extracted to infer site-level m6A probabilities using a neural network model. Outputs include motif enrichment, metagene profiles, biotype distributions, and group-level m6A comparisons.

Supplementary Figure S2

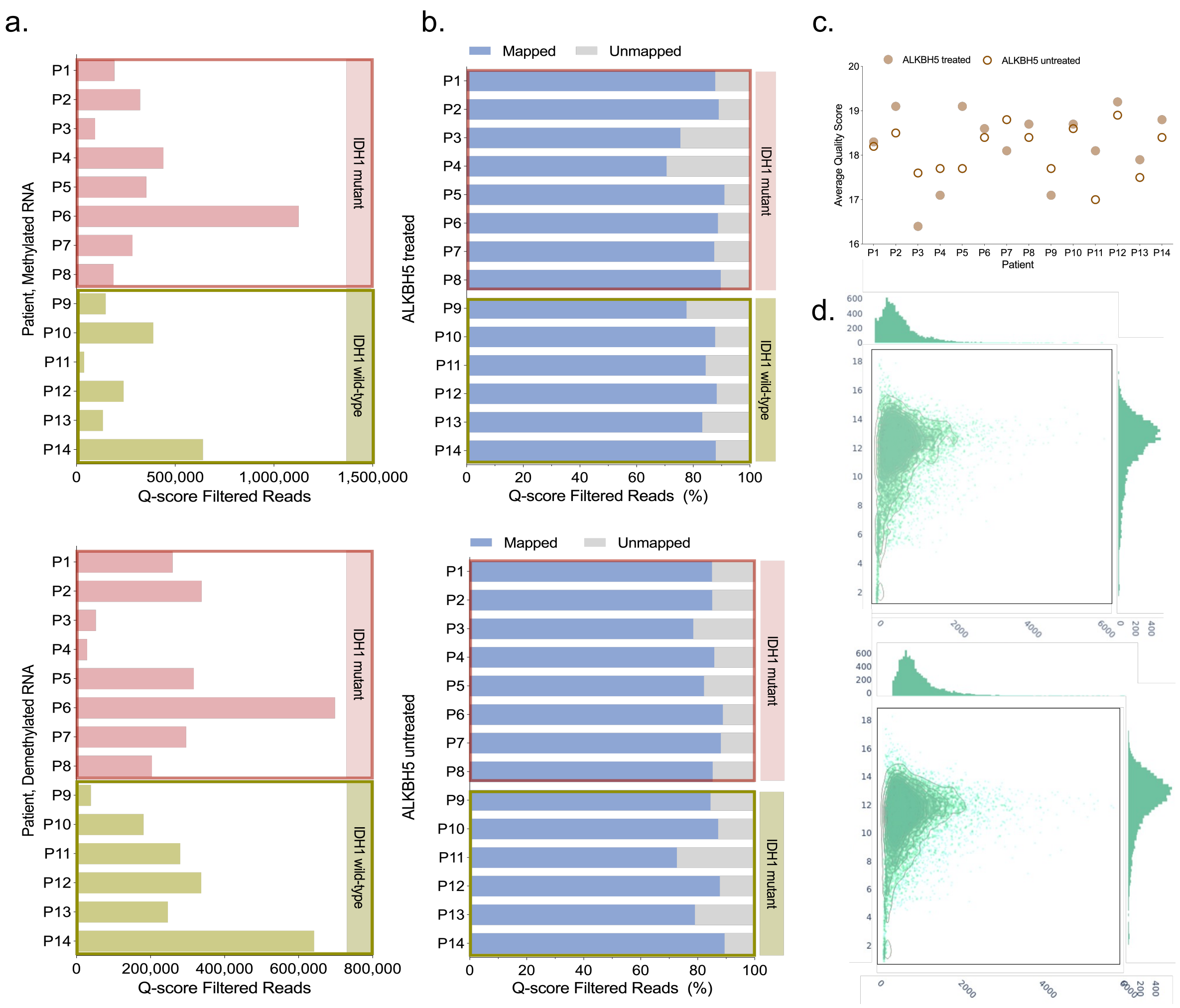

Supplementary Figure S2. Comparison of sequencing output in ALKBH5 treated and untreated RNA. (a) Bar graphs depicting the number of Q score filtered reads in ALKBH5 treated (top) and untreated (bottom) RNA sequenced from individual patients. (b) Distribution of mapped and unmapped reads during alignment for ALKBH5 treated (top) and untreated (bottom) samples. (c) Dot plot comparing the the average quality score of the sequencing output from treated and untreated RNA in individual patients. (d) Quality control (QC) Nanoplots plotting the log transformed read lengths against average read quality (using a kernel density estimate) in ALKBH5 treated (top) and untreated (bottom) samples.

Supplementary Figure S3

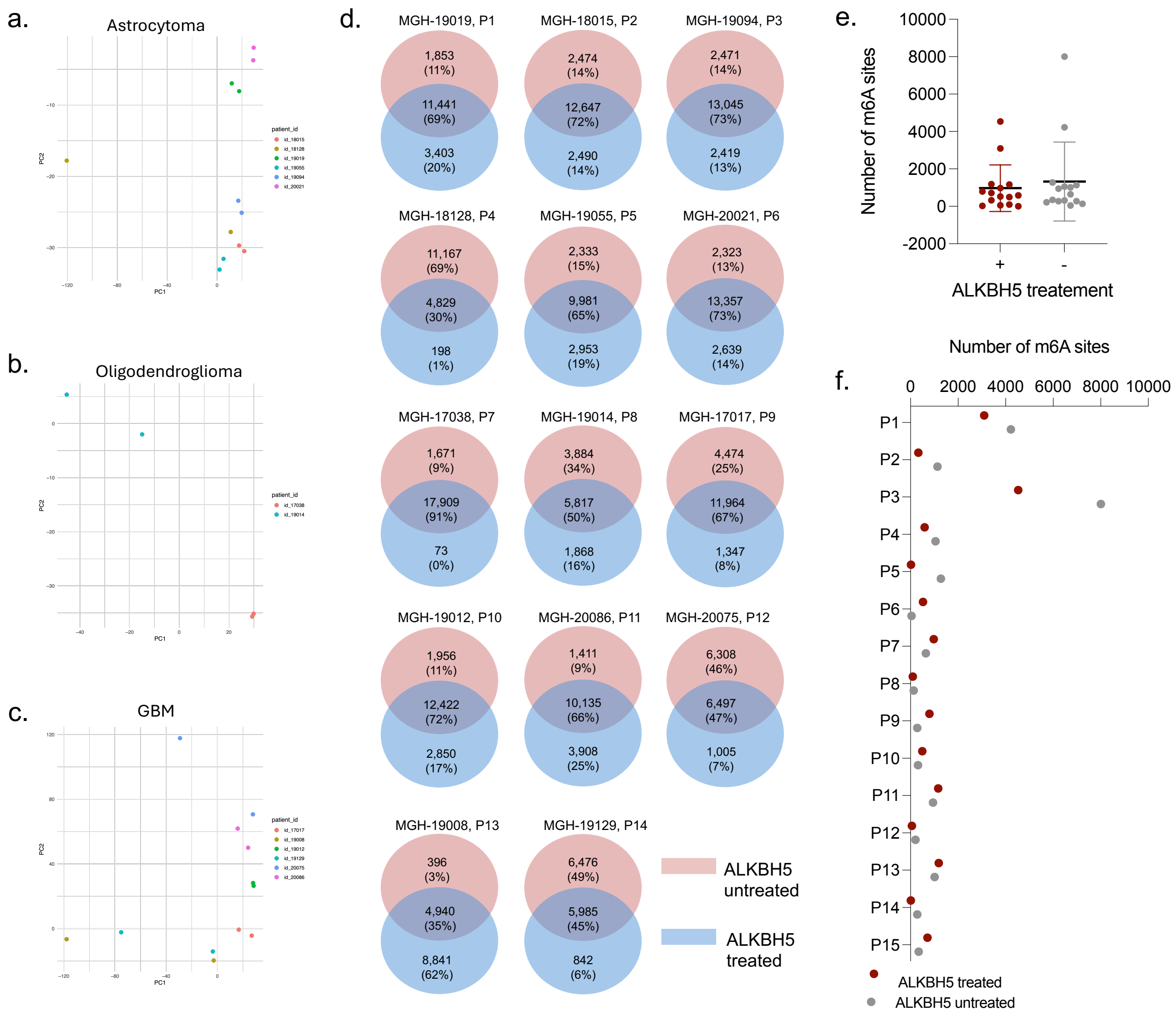

Supplementary Figure S3. Comparison of gene expression and m6A site detection with ALKBH5 treatment. (a-c) PCA plots highlighting the clusters of gene expression data for ALKBH5 treated and untreated RNA in (a) AA, (b) OO, and (c) GBM. (d) Venn diagrams demonstrating the number and percentage of common and unique genes detected in ALKBH5 treated and untreated RNA across individual patients. (e) Distribution of detected high confidence (probability modified > 0.9) m6A sites between ALKBH5 treated (red) and untreated RNA (gray) with annotated median and SD. (f) Distribution of detected high confidence) m6A sites between ALKBH5 treated (red) and untreated RNA (gray) across individual patients.

Supplementary Figure S4

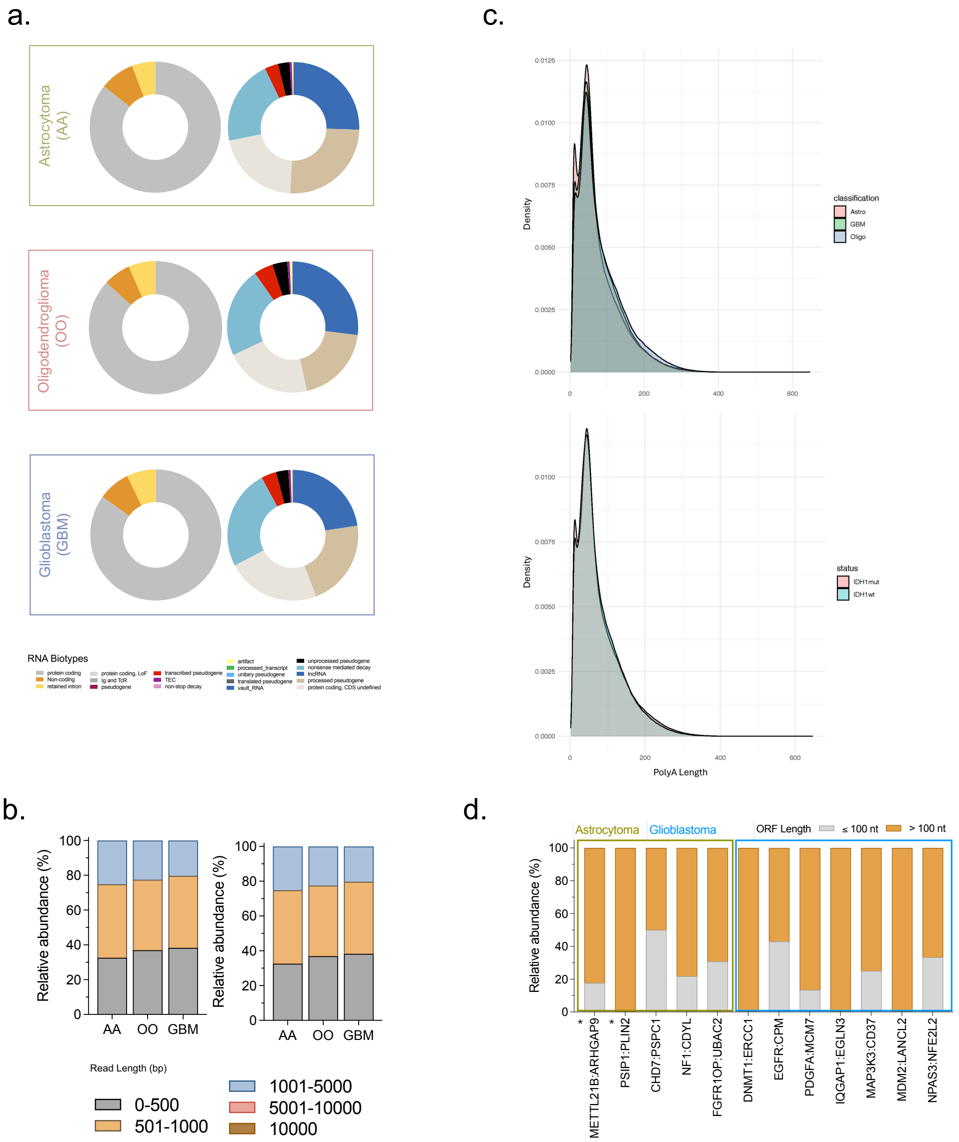

Supplementary Figure S4. Distributions of key transcriptomic features across glioma subtypes. (a) Pie charts showing the distribution of RNA biotypes across glioma subtypes. The left panel displays the proportions of protein-coding, retained intron, and non-coding transcripts. The right panel further breaks down the composition of the non-coding transcript category. (b) Stacked bar chart showing the relative abundance (%) of mapped read lengths of overall (left) and full-length (right) annotated transcripts across subtypes. (c) Poly-a tail density distribution across (top) AA, OO, and GBM and (bottom) IDH1 mutant and IDH1 wild-type. (d) Relative abundance and ORF length distribution of detected fusion transcripts in AA and GBM. Asterisks denotes high confidence fusion transcripts.

Supplementary Figure S5

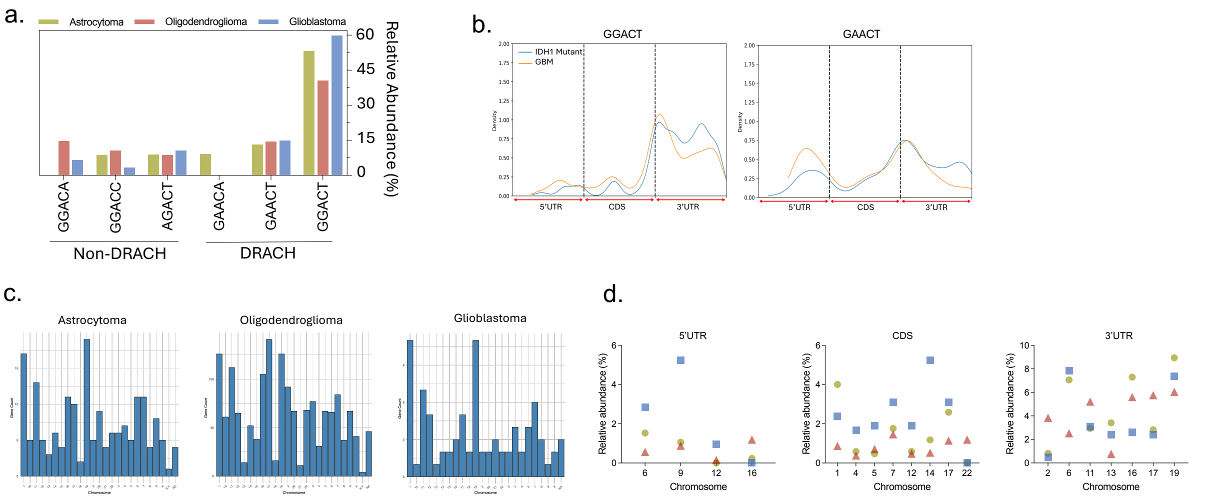

Supplementary Figure S5. Transcriptomic analysis of detected m6A modified sites. (a) Distribution of the top 6 DRACH and non-DRACH Kmers across the glioma subtypes. (b) KDE plots representing the distribution of the top 2 m6A kmers along the transcript regions (5'UTR, CDS, and 3'UTR) across the glioma subtypes. (c) Chromosomal distribution of the filtered m6A sites (probability of modification ≥ 0.90), highlighting the frequency of modified genes per chromosome across three glioma subtypes. (d) Scatter plots demonstrating the spatial relative distribution of m6A modified genes in Astrocytoma (green circle), Oligodendroglioma (red triangle) and Glioblastoma (blue square) across the distinct transcript regions: 5’UTR (left), CDS (middle), and 3’UTR (right).

Supplementary Figure S6

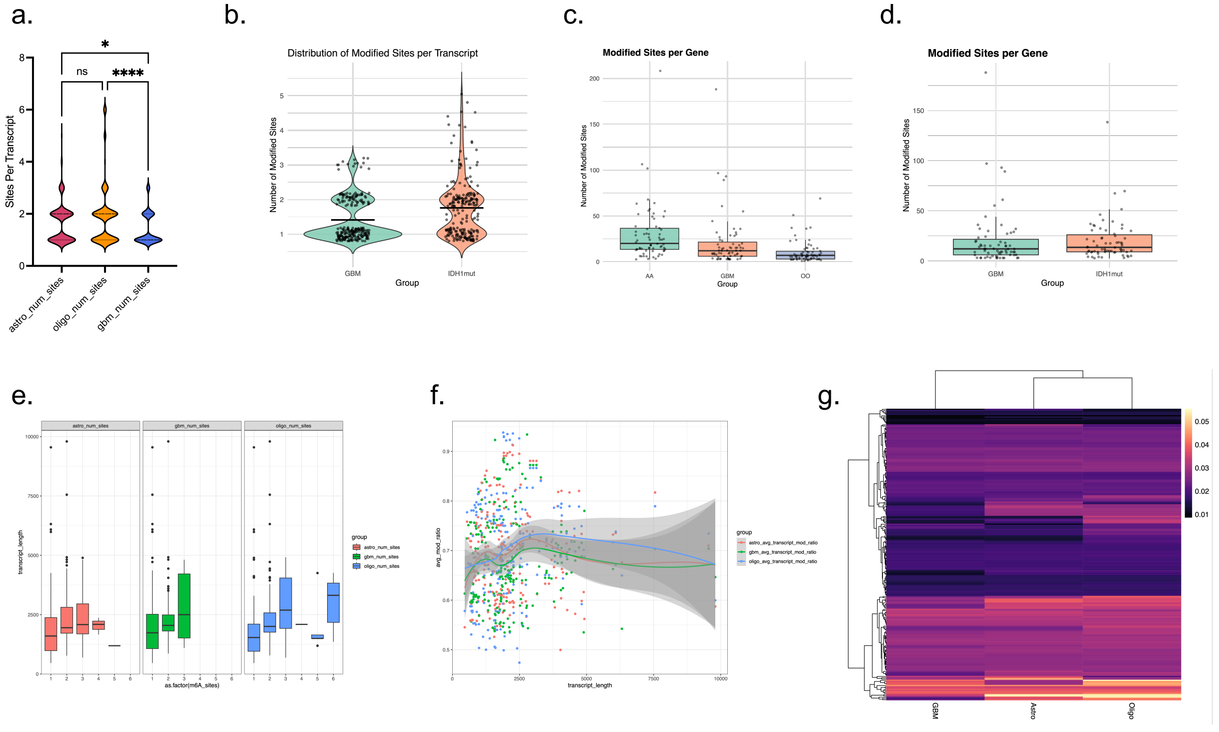

h.

Upregulated Gene Group (rows: Hypermethylated Group)

Hypermethylated Group	AA	GBM
AA	EEF1A1 (16)<br>LDHB (3)	ACTB (6)<br>AP2S1 (5)<br>LGALS3BP (4)<br>ATP6V0E1 (2)<br>S100B (2)<br>CEBPD (1)<br>CENPB (1)
GBM	BCAN (1)<br>EEF1A1 (1)<br>EEF2 (1)	HSPA5 (6)<br>ACTB (4)<br>APOD (2)<br>TMSB10 (1)<br>SPP1 (1)

Upregulated Gene Group (rows: Hypermethylated Group)

Hypermethylated Group	OO	GBM
OO		APS21 (5)<br>HSPA5 (5)<br>TUBB2A (5)<br>LGALS3BP (4)<br>DKK3 (3)<br>S100B (2)<br>CENPB (1)
GBM	BCAN (1)	DYNLL1 (4)<br>C1orf122 (3)<br>CEBPD (1)<br>TMSB10 (1)<br>HSPA5 (1)

Upregulated Gene Group (rows: Hypermethylated Group)

Hypermethylated Group	AA	OO
AA	TUBB2A (4)<br>C1orf122 (3)<br>CEBPD (1)	C1QA (8)<br>HTRA1 (1)
OO	DKK3 (3)<br>TUBB2A (1)	HTRA1 (2)

Supplementary Figure S6. Commonly modified transcripts across the glioma subtypes demonstrate variation in m6A levels. (a) Violin plots depicting the distribution of sites per transcript for the commonly methylated transcripts. A significant difference was observed among AA (red), OO (orange), and GBM samples (blue) (Kruskal-Wallis test, $p < .05$). (b) Violin plots depicting the modified sites per transcript GBM (green, mean = 1.41) and IDH1 mutant (orange, mean = 1.76), with a significant difference between groups (Wilcoxon rank sum test, $p < .05$). (c) Box plots showing the number of modified sites per gene. There was no significant difference between groups. (e) Box plots showing the number of modified sites per gene in GBM (green) and IDH1 mutant (orange). There was no significant difference between groups. (e) Boxplots showing the distribution of transcript length (y-axis) across different numbers of m6A sites per transcript (x-axis) for AA (red), GBM cells (green), and OO (blue). (f) Distribution of average modification ratio per site (y-axis) across transcript length (x-axis) per classification. (g) Heatmap illustrating the distribution of the calculated weighted modification ratio for commonly

m6A-methylated transcripts across all classifications. A square root transformation was applied to enhance the visualization of differences in modification levels. (h) Upregulated genes and hypermethylated gene groups in different sample comparisons (AA vs. GBM, left; OO vs. GBM, middle; AA vs. OO, right), with numbers indicating the number of transcripts for each gene within each overlapping category.

Supplementary Figure S7

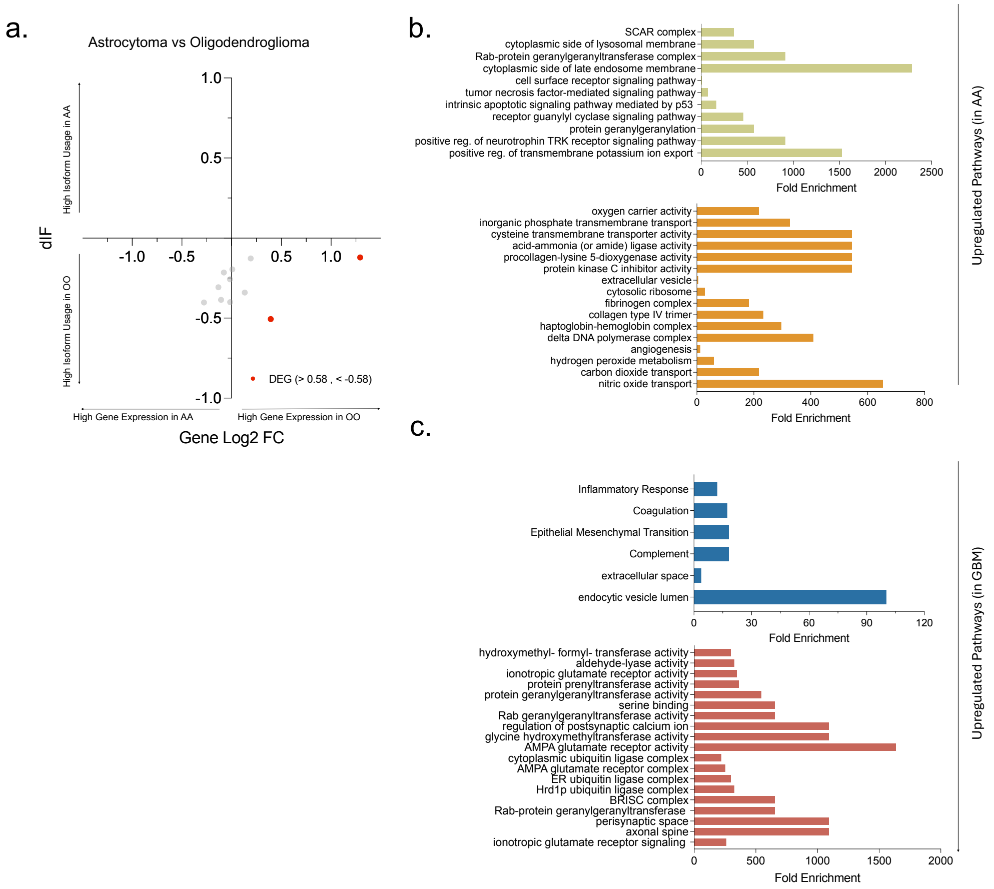

Supplementary Figure S7. Isoform Switching Analysis with Gene Expression and Functional Pathways (a) Scatter plot of differences in isoform fraction (dIF, y-axis) and differential gene expression (Log2 FC, x-axis) for AA vs OO comparison, with significant points highlighted in red. (b) Gene ontology (GO) analysis of significantly upregulated genes in AA (top) and GBM (bottom) to identify upregulated pathways. Fold enrichment plotted for significantly enriched pathways. (c) GO analysis of significantly downregulated genes in AA (top) and GBM (bottom) to identify downregulated pathways. Fold enrichment plotted for significantly enriched pathways.

Supplementary Figure S8

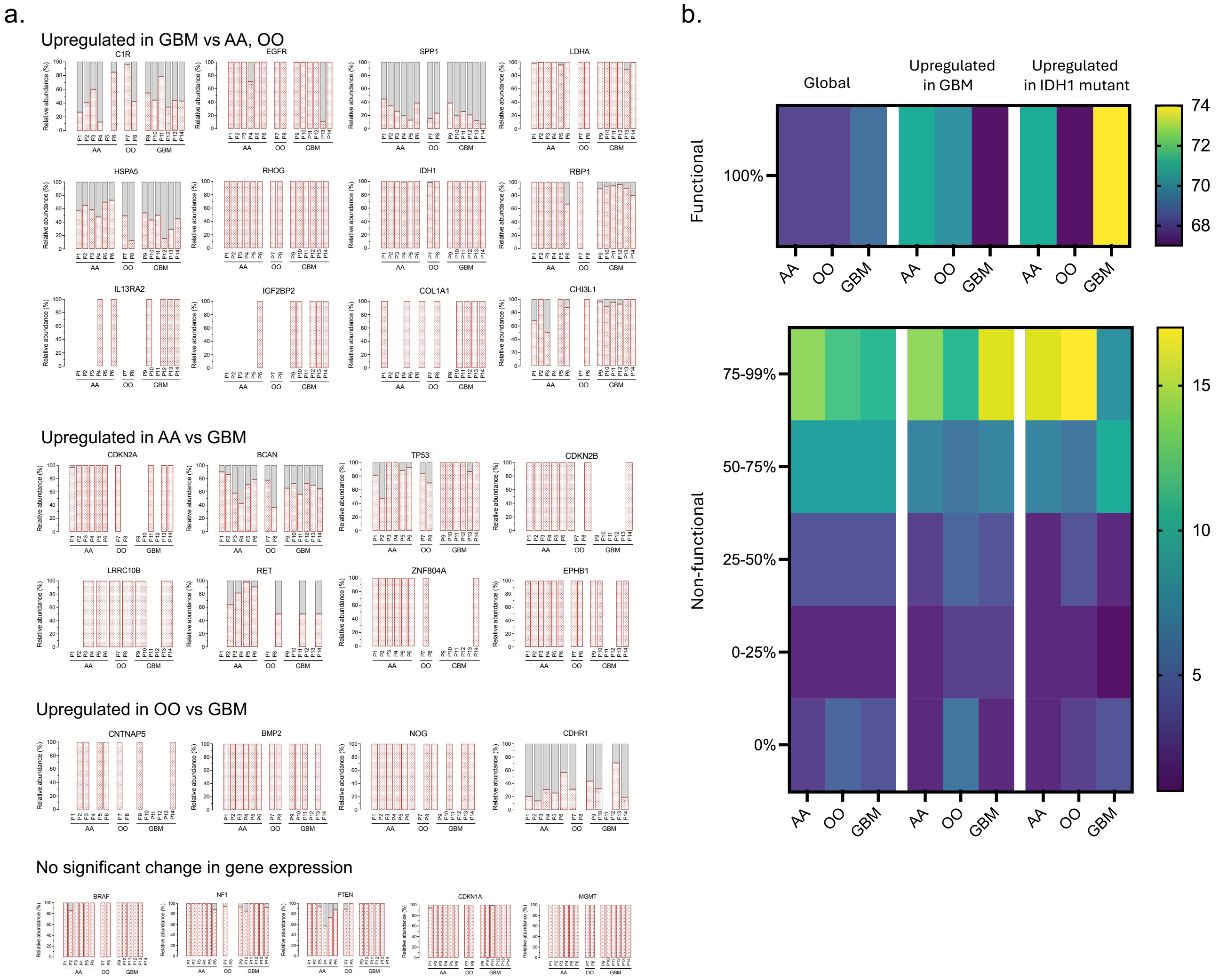

Supplementary Figure S8. Analysis of functional and non-functional transcript composition among protein-coding genes. (a) Relative abundance of functional and non-functional transcripts per gene across patients. Protein-coding genes were stratified based on differential expression: genes upregulated in GBM compared to AA and OO, genes upregulated in AA versus GBM, genes upregulated in OO versus GBM, and genes with no significant differential expression. (b) Heatmap showing the percentage distribution of genes containing 0–100% functional transcripts. The left panel includes all expressed genes, the middle panel highlights genes upregulated in GBM relative to IDH1 mutant gliomas, and the right panel shows genes upregulated in IDH1 mutant gliomas compared to GBM.

Supplementary Table S1

Label	IDH status	Classification	WHO Grade	Age when Enrolled	Gender	Extent of Resection	Location	Recurrence	MGMT status	IDH variant	1p/19q	EGFR	EGFRvIII	TERT	TP53	Deceased	Overall Survival (Days)	Progression Free Survival (Days)
P1	mut	Astrocytoma	2	39	M	STR	Frontoparietal	no	unmethylated	R.132H	N/A	wt	wt	wt	mut	no	2194	2194
P2	mut	Astrocytoma	3	30	F	GTR	Frontal	no	unmethylated	R.132H	maintained	wt	wt	wt	mut	no	2583	2583
P3	mut	Astrocytoma	3	37	M	GTR	Frontal	no	unmethylated	R.132H	N/A	wt	wt	wt	mut	no	2038	2038
P4	mut	Astrocytoma	2	44	F	STR	Frontal	yes	unmethylated	R.132H	N/A	wt	wt	wt	mut	yes	1512	1207
P5	mut	Astrocytoma	3	29	F	GTR	Temporal	no	methylated	R.132H	N/A	wt	wt	wt	mut	no	2084	2084
P6	mut	Astrocytoma	4	34	M	STR	Temporal	yes	unmethylated	R.132H	N/A	wt	wt	wt	mut	yes	1578	772
P7	mut	Oligodendroglioma	2	40	F	NTR	Frontal	no	methylated	R.132H	co-deleted	wt	wt	wt	wt	no	2649	2649
P8	mut	Oligodendroglioma	2	26	F	GTR	emporo-parieta	yes	methylated	R.132G	co-deleted	wt	wt	wt	wt	no	2200	946
P9	wt	GBM	3	66	F	GTR	Frontal	yes	unmethylated	NA	N/A	amplified	mut	mut	wt	yes	445	355
P10	wt	GBM	4	60	F	NTR	Frontal	yes	unmethylated	NA	N/A	amplified	mut	mut	wt	yes	785	391
P11	wt	GBM	4	64	F	GTR	Frontal	yes	methylated	NA	N/A	amplified	wt	mut	wt	yes	1279	1029
P12	wt	GBM	4	49	M	GTR	Temporal	yes	methylated	NA	N/A	wt	wt	mut	wt	yes	1236	772
P13	wt	GBM	2	87	M	NTR	Frontal	no	unmethylated	NA	N/A	wt	N/A	N/A	N/A	yes	462	462
P14	wt	GBM	1	27	M	GTR	cerebellum	no	unmethylated	NA	N/A	N/A	N/A	N/A	wt	no	1877	1877

Supplementary Table S1. Clinical characteristics for patient cohort.

Supplementary Table S2

Cluster 1	Cluster 2	Cluster 3	Cluster 4
ZNF561-AS1	MIR9-1HG	SSH3	NFIA-AS2
FGFR1	SNHG29	POLD1	MIR497HG
IFNAR1	CDIP1	PLOD1	SHMT2
MRPL36	RABGGTB	ZNF224	LRIG1
PLOD1	CYFIP2	NNMT	GRIA4
MRE11	NPPA	SLC20A2	GRIA1
CTSL		MFSD12	OS9
C1S		COL4A1	RABGGTB
SERPINA1		TMEM176B	CD27-AS1
LGI4		FN1	WDR45
ITGA7		HSPB1	
ITGA7		HBA1	
LY96		GLUL	
RNASE4			
PDLIM4			
TRAPPC13			
EMP3			
HSPB1			
HBA1			
CHI3L1			

Supplementary Table S2. Gene lists for isoform switching analysis clusters in the astrocytoma (AA) versus glioblastoma (GBM) comparison (Figure 4c). Cluster 1: higher isoform usage in AA and higher gene expression in GBM. Cluster 2: higher isoform usage and expression in AA. Cluster 3: higher isoform usage and expression in GBM. Cluster 4: higher isoform usage in GBM and higher gene expression in AA.
